# Natural Molecules for Brain Health and Resilience

**DOI:** 10.3390/ijms27104343

**Published:** 2026-05-13

**Authors:** Vasiliki Venetsanaki, Eleni C. Pardali, Christos Cholevas, Maria G. Grammatikopoulou, Dimitrios G. Goulis, Theoharis C. Theoharides

**Affiliations:** 1 Unit of Reproductive Endocrinology, First Department of Obstetrics and Gynecology, Medical School, Aristotle University of Thessaloniki, GR-54124 Thessaloniki, Greece; venetsanaki.v@gmail.com (V.V.); dgg@auth.gr (D.G.G.); 2 Immunonutrition Unit, Department of Rheumatology and Clinical Immunology, Faculty of Medicine, School of Health Sciences, University of Thessaly, Biopolis, GR-41223 Larissa, Greece; elpardali@uth.gr (E.C.P.);; 3 Department of Clinical Pharmacology, Faculty of Medicine, Aristotle University of Thessaloniki, GR-54124 Thessaloniki, Greece; ccholevas@auth.gr; 4 Institute for Neuro-Immune Medicine, Nova Southeastern University, Clearwater, FL 33759, USA; 5 Department of Immunology, Tufts University School of Medicine, Boston, MA 02111, USA

**Keywords:** dietary supplements, brain, biotin, flavonoids, folic acid, huperzine A, *Hericium erinaceus*, luteolin, oleuropein, hydroxytyrosol, palmitoylethanolamide, neuronutrition

## Abstract

The global rise in cognitive decline and neurodegenerative disorders has intensified the search for safe and accessible strategies to support brain health. In recent years, nutraceuticals have gained considerable attention as potential modulators of neurological function due to their antioxidant, anti-inflammatory, and neuroprotective properties. Increasing evidence suggests that oxidative stress, neuroinflammation, mitochondrial dysfunction, and impaired neurovascular integrity play central roles in the pathogenesis of several neurodegenerative diseases, namely Alzheimer’s, Parkinson’s disease and autism spectrum disorder, among others. This narrative review provides an integrated overview of selected nutraceuticals with potential relevance to brain-related disorders, including biotin, folinic acid, flavonoids (apigenin, diosmin, luteolin, naringin, pycnogenol, and quercetin), huperzine A, Lion’s mane, olive oil polyphenols, oleuropein and palmitoylethanolamide. Rather than presenting a purely descriptive summary, we considered both mechanistic and clinical evidence, highlighting differences in the strength, consistency, and quality of available data across compounds. Among the reviewed compounds, huperzine A, specific flavonoids—particularly luteolin—and olive oil polyphenols demonstrated relatively stronger and more consistent support across experimental models and emerging clinical studies, mainly through modulation of cholinergic signaling, neuroinflammatory pathways, and oxidative stress responses. In contrast, evidence for other agents remains limited, heterogeneous, or primarily at the preclinical level. Overall, this review aims to provide a clearer and more structured synthesis of the current literature on neuronutrition, identifying compounds with the most promising profiles while outlining key limitations and research gaps that need to be addressed to better define their role in brain health.

## 1. Introduction

Brain health is a multidimensional and evolving concept that is difficult to evaluate and quantify [1]. In a recent position paper, the World Health Organization (WHO) defined brain health as a state of brain functioning across cognitive, sensory, social–emotional, behavioral, and motor domains, allowing a person to realize their full potential over the life course, irrespective of the presence or absence of disorders [1].

Neurological conditions, including stroke, dementia, epilepsy, injuries, tumors, infections, neurodevelopmental disorders, and congenital conditions, are among the leading causes of global mortality and disability-adjusted life years (DALYs), with over three billion people affected worldwide [2,3]. In Europe, the total costs of neurological disorders in the year 2020 were estimated to be 1.7 trillion €, which is more than the combined costs for treating cardiovascular disease (CVD), cancer, and diabetes mellitus, together [4]. The WHO has recognized this as a public health crisis and has launched an Intersectoral Global Action Plan on Epilepsy and Other Neurological Disorders (IGAP), with three main global targets: (i) to reduce the stigma, impact, and burden of neurological disorders; (ii) to optimize neurological and brain health across the life course; and (iii) to improve the quality of life of affected people, their families, and caregivers [5].

To date, there has been no adequate action towards effective and equal access to services, support, medicines, and diagnostics. Modifiable determinants known to impact brain health across the life span include physical activity, diet, tobacco and alcohol use, cognitive activity, and metabolic factors. Nutritional deficiencies have been linked to the onset of cognitive decline and dementia in later life [6,7]. In contrast, healthy, balanced diets and healthy body weight management have been recognized as protective factors against the development of neurological diseases [5].

Treatment options have evolved, and breakthroughs in gene therapy for spinal muscular atrophy in children have revolutionized the treatment of neurological diseases; however, the majority of available treatments for neurological diseases provide only symptom relief, and not prevention or a cure [8]. Diet has again come to the rescue, and several dietary patterns and components have been investigated with regard to brain health [9,10]. Research in the field of psycho-neuro-endocrino-immunology (PNEI) has expanded our understanding of how nutrition influences the complex interactions between the mind, hormones, and the immune system [11]. Neuronutrition is the interdisciplinary area of nutritional neuroscience, studying the effects of diet, nutrients and dietary components on brain health, behavior and cognition [11]. It also aims in improving brain resilience to stressors, while also prevent, treat, and neuro-rehabilitate cognitive function, behavioral or neurological disorders across the lifespan, using the science of nutrition [12,13].

Among the dietary treatments examined, nutraceuticals have emerged as potential modulators of brain health [14]. Several bioactive compounds derived from foods and natural sources exhibit antioxidant, anti-inflammatory, and neuromodulatory properties that may contribute to neuroprotection [14,15]. Oxidative stress and neuroinflammation are recognized as key contributors to the pathogenesis of neurodegenerative disorders, including Alzheimer’s disease (AD) [16,17]. In particular, oxidative stress can damage the neurovascular unit and cerebral endothelium, leading to impaired cerebral blood flow and hypoperfusion, which are commonly observed in AD [18]. Moreover, excessive production of reactive oxygen species (ROS) has been implicated in amyloid-β (Aβ) aggregation and plaque formation, further promoting neuronal dysfunction and neurodegeneration [16,19,20].

**Figure 1:**
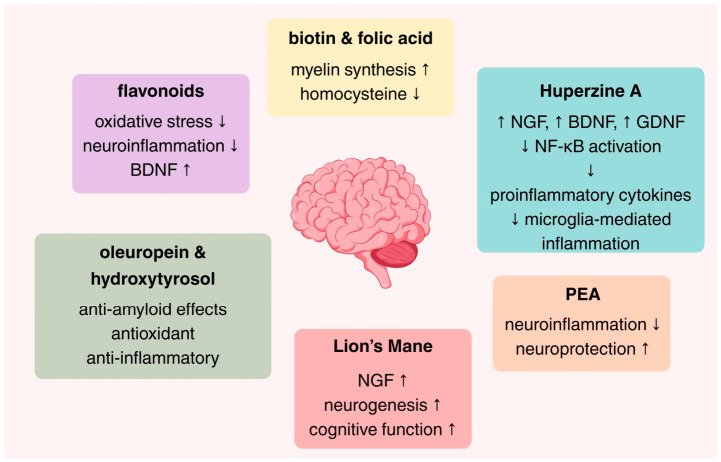
Mechanisms of Action of Select Nutraceuticals in Neuroprotection and Cognitive Function. BDNF: brain-derived neurotrophic factor; NGF: nerve growth factor; GDNF: glial cell line-derived neurotrophic factor; NF-κB: Nuclear Factor Kappa B; PEA: palmitoylethanolamide. ↑: increased; ↓: decreased.

Thus, in the present review we aimed to summarize the preclinical and clinical evidence of selected nutraceuticals, focusing on biotin, flavonoids especially luteolin, folic acid, Huperzine A, Lion’s mane, olive oil polyphenols (oleuropein and hydroxytyrosol [HT]), and palmitoylethanolamide (PEA). The present narrative review highlights current mechanistic insights, as well as evidence from experimental and clinical studies regarding their potential roles in supporting cognitive function, modulating neuroinflammation, and mitigating neurodegenerative processes. It should be noted that the present work consists of a review and not a dosing guideline. The mechanisms underlying these effects are presented in Figure 1.

## 2. Mechanistic Overview of Nutraceutical Effects on Brain Health

Neurodegenerative processes are driven by a complex interplay of oxidative stress, neuroinflammation, and mitochondrial dysfunction, which together form a central pathogenic triad [21]. Excessive production of reactive oxygen species (ROS) damages lipids, proteins, and nucleic acids, compromising neuronal integrity and disrupting signaling pathways involved in cell survival, synaptic plasticity, and neurogenesis [22]. Concurrently, chronic activation of microglia and astrocytes establishes a persistent pro-inflammatory milieu through the sustained release of cytokines and chemokines [including tumor necrosis factor-α (TNF-α), interleukin 1α/β (IL-1α/β), and interleukin-6 (IL-6)], which further amplifies oxidative damage and promotes apoptosis [23,24]. Mitochondrial dysfunction exacerbates these processes by reducing adenosine triphosphate (ATP) production, disturbing Calcium homeostasis, and triggering intrinsic apoptotic pathways, while also intensifying oxidative stress and inflammatory signaling [21,25]. Nutraceuticals can intervene across this pathogenic network through multiple mechanisms [14,15]. Bioactive polyphenols, including flavonoids and olive oil derivatives, modulate redox-sensitive transcription factors, such as the Nuclear factor erythroid 2-related factor (NRF2), enhancing endogenous antioxidant enzyme expression while simultaneously inhibiting pro-inflammatory pathways, including the nuclear factor kappa-light-chain-enhancer of activated B cells (NF-κB) and the NOD-, LRR- and pyrin domain-containing protein 3 (NLRP3) inflammasome [26,27,28,29,30,31].

Recent mechanistic evidence further supports that flavonoid-mediated neuroprotection is highly structure- and subclass-dependent, with distinct compounds targeting specific intracellular signaling cascades rather than exerting uniform antioxidant effects [32]. Emerging flavonoids, including glycosylated derivatives such as prunin, have been shown to modulate phosphatidylinositol 3-kinase (PI3K)/protein kinase B (Akt) and mitogen-activated protein kinase (MAPK) signaling pathways, directly influencing cell survival, proliferation, and stress responses [32]. This pathway is critically involved in the regulation of mitochondrial apoptosis, where its modulation influences the balance between pro-apoptotic (e.g., Bax, Bad) and anti-apoptotic [e.g., B-cell lymphoma 2 (Bcl-2)] proteins, ultimately determining mitochondrial membrane permeability and cytochrome c release [32,33,34,35]. In parallel, flavonoids influence ROS-dependent signaling networks, not only by reducing oxidative burden but also by regulating redox-sensitive kinases such as c-Jun N-terminal kinases (JNK) and p38 MAPK, which are critical mediators of stress-induced apoptosis [32].

In addition to flavonoids, other nutraceutical classes contribute to this integrated network through complementary mechanisms. Vitamins and metabolic co-factors, such as folic acid and biotin, support mitochondrial metabolism, methylation pathways, and genomic stability, thereby indirectly influencing the redox balance and neuronal function [36,37,38,39,40]. Lipid-derived mediators, including PEA, exert anti-inflammatory effects through nuclear receptor signaling (e.g., PPAR-α), attenuating microglial activation and cytokine release [41,42,43]. Neuroactive compounds, such as Huperzine A and bioactive compounds from Lion’s mane, further enhance neurotrophic signaling pathways, including brain-derived neurotrophic factor (BDNF)- and PI3K/Akt-mediated cascades, supporting synaptic plasticity and neuronal survival [44,45,46].

Collectively, these nutraceuticals act on primary mechanistic drivers, while downstream effects enhance synaptic function, neurotransmission, neurogenesis, and proteostasis. By targeting multiple, interconnected pathways, they exert synergistic effects that reinforce brain resilience, slow neurodegenerative progression, and maintain cognitive function. This integrated mechanistic framework provides a foundation for understanding the specific roles of each nutraceutical discussed in the following sections, highlighting how distinct molecular actions converge on shared protective networks.

## 3. Biotin

### 3.1. Introduction

Biotin (vitamin B7), a water-soluble B-complex vitamin, is essential for cellular metabolism because it serves as a cofactor for the activation of carboxylases involved in gluconeogenesis and the metabolism of amino acids and fatty acids. Biotin (cis-hexahydro-2-oxo-1H-thieno [3,4-d] imidazole-4-pentanoic acid) is composed of two organic rings. One ring contains a ureido group and is involved in non-covalent binding to avidin, and the other ring contains a tetrahydrothiophene group with a valeric acid moiety attached as a side chain. Biotin can be covalently attached to a variety of proteins to exert cellular functions [47]. The four biotin-dependent carboxylases are acetyl-CoA carboxylase (ACC) 1 and 2, methylcrotonyl-CoA carboxylase (MCC), propionyl-CoA carboxylase (PCC), and pyruvate carboxylase (PC) [15]. Biotin binds to the biotin carboxyl carrier protein (BCCP) domain of each enzyme. This process is catalyzed by holocarboxylase synthetase (HLCS), which attaches biotin to a lysine residue, forming biocytin. Biotin carboxylase then catalyzes the transfer of carbon dioxide from bicarbonate to biocytin affixed to BCCP, and the carboxyltransferase domain transfers the carboxyl group from biotin to various acceptor molecules, forming new compounds that participate in important metabolic pathways [15]. ACC is crucial for the synthesis of fatty acids, MCC for the catabolism of leucine, PC is involved in the first step of gluconeogenesis, and PCC catabolizes amino acids, odd-chain fatty acids, and the side chain of cholesterol [47].

In addition to its cellular functions, biotin may affect cell proliferation, deoxyribonucleic acid (DNA) repair, and the expression of over 2000 genes by virtue of its role in histone biotinylation in the cell nucleus; however, the exact mechanism is still under investigation [47,48].

Mammals cannot synthesize biotin, which can only be obtained through diet or from the human gut microbiota. Inadequate dietary intake is rare because biotin is present in a variety of food items; dietary sources of biotin include meat, egg yolks, dairy products, nutritional yeast, vegetables, peanuts, soybeans, sunflower seeds, mushrooms, and sweet potatoes. Protein-bound biotin is digested by proteases and peptidases in the gastrointestinal system into biotinylated oligopeptides and biocytin, which are then converted to free biotin before intestinal absorption by biotinidase [49]. Diet-obtained biotin is absorbed in the small intestine, but gut microbiota-generated biotin largely depends on the composition of gut microbiota and is absorbed from the large intestine.

The recommended daily intake for adults and pregnant women is 30 and 35 μg/d for lactating women, respectively. Factors that inhibit biotin absorption or increase biotin catabolism include alcohol abuse, smoking, inflammatory bowel disease, and anticonvulsant medications [48]. Decreased microbial biosynthesis of biotin has been reported in patients with obesity, type 1 diabetes mellitus, isolated systolic hypertension and insomnia [47].

The importance of biotin in the function of the nervous system is highlighted by inherited disorders associated with biotin deficiency. Genetic deficiencies in HLCS and biotinidase can cause biotin deficiency. Holocarboxylase synthetase deficiency is an autosomal recessive disorder that results in defective biotinylation. The clinical syndrome includes neurological symptoms, such as hypotonia, seizures, difficulty in breathing and feeding, metabolic acidosis, hyperammonemia, organic aciduria, skin rash, and alopecia. Biotinidase deficiency is another autosomal recessive disorder that results in a defect in biotin recycling. Neurological symptoms predominate, along with visual and hearing disturbances, metabolic acidosis, organic aciduria, and cutaneous and immunological disorders. Treatment with pharmacological doses of free biotin has been used to treat affected children [49].

Biotin-responsive basal ganglia disease (BBGD) is an orphan neurometabolic disease caused by mutations in *SLC19A3*, which encodes a thiamine transporter. Patients with BBGD may develop subacute encephalopathy, dysarthria, dysphagia, and nerve palsies, progressing to permanent disability and even death if left untreated. Treatment with high doses of biotin (5–10 mg/kg/day) and thiamine dramatically improves symptoms; however, upon discontinuation, the symptoms recur [49,50].

In reality, biotin supplements are marketed towards the general public rather than patients with these rare disorders. Biotin supplementation to promote skin, hair, and nail health has become very popular, even among healthy individuals. In fact, health care practitioners also recommend biotin treatment to address skin rashes, hair regrowth, and brittle nails. In preclinical studies, a lack of biotin in the diet of rats caused neuromuscular dysfunction, alopecia, and dermatitis. In clinical practice, most claims on the effectiveness of biotin in dermatological conditions are based only on case reports of biotin-deficient children with atopic dermatitis. The effectiveness of oral biotin for brittle nails has been better studied [51,52]. Biotin popularity despite the significant lack of evidence could be attributed to the low cost, over-the-counter availability, and lack of toxicity even in larger doses. Indeed, toxicity from high intake has not been reported. However, it is now known that biotin may interfere with laboratory immunoassays that are based on biotin–(strept)avidin technology and affect the results of troponin, pro-brain natriuretic peptide, thyroid-stimulating hormone, free triiodothyronine, and parathyroid hormone. Biotin can either cause falsely increased concentration values if a competitive assay is used (positive interference) or falsely reduced concentrations if a sandwich immunoassay is used (negative interference). Based on pharmacokinetic data, it is recommended to discontinue treatment for 8 h to 7 days, depending on the dose of biotin treatment [51].

### 3.2. Preclinical Studies

Preclinical evidence suggests that biotin homeostasis may be prioritized in the central nervous system (CNS). In a preclinical study, biotin utilization from the liver was downregulated when biotin availability was reduced to ensure a continued supply of biotin to the brain [53]. A few studies have also investigated biotin in models of neurodegenerative diseases. In cultured human dopaminergic neurons, biotin supplementation exhibited a protective effect against manganese-induced mitochondrial dysregulation, cytotoxicity, and neuronal loss. Biotin supplementation rescued mitochondrial deficits and improved neuronal health [54]. In an AD rodent model, Almasi et al. showed that biotin supplementation improved Aβ-induced memory impairment and attenuated oxidative stress markers [55]. In an adult fly model of manganese-induced Parkinson’s disease (PD), Lai et al. identified systemic changes in biotin metabolism. Manganese-induced neurotoxicity, parkinsonism, and mitochondrial dysfunction were exacerbated in biotinidase-deficient flies, and biotin supplementation mitigated these neurotoxic effects. In cultured human dopaminergic neurons, biotin supplementation exhibited a protective effect against manganese-induced mitochondrial dysregulation, cytotoxicity, and neuronal loss [56].

High doses of biotin have been extensively studied in patients with multiple sclerosis (MS); however, preclinical evidence remains scarce. The premise for its use is based on the observation that in oligodendrocytes, biotin enhances myelin synthesis by binding to ACC and increasing the availability of fatty acids. In neurons, biotin reduces hypoxia by binding to enzymes necessary for the formation of tricarboxylic acid cycle intermediates and increasing ATP production. These findings support the notion that biotin may induce myelin repair and reduce hypoxia-induced axonal degeneration among patients with MS [57,58].

One animal study investigated the effect of different doses of biotin on brain damage after exposure to radiation. Administration of 6 mg biotin reduced markers of oxidative stress, inflammation, and apoptosis. Although preliminary, these findings support further research on the prevention of radiation-induced brain injury in patients treated with radiotherapy for certain malignancies [59].

### 3.3. Clinical Studies

Over the past decade, high-dose biotin has received clinical interest as a potential treatment approach for progressive MS (Table 1). This is based on the premise that high-dose biotin can increase energy production in order to reduce hypoxia and protect against neuronal degeneration or enhance myelin repair through the activation of biotin-dependent carboxylases [60]. MS is one of the most common, disabling neurological diseases that affects young adults. Its clinical course usually involves an initial phase of relapsing–remitting (RRMS) bouts, later evolving to a secondary progressive disease (SPMS). Primary progressive MS (PPMS) with disease progression on onset is less common. During the progressive phase, patients experience pain, changes in vision, walking and balance, combined with cognitive changes. Disease-modifying therapies primarily target inflammation to reduce exacerbations and delay progression include immunomodulators, pyrimidine synthesis inhibitors, fumarates, sphingosine-1-phosphate receptor modulators, monoclonal antibodies, and other agents.

In an initial pilot study including 23 patients with PPMS or SPMS, patients were treated with high-dose biotin for 2–36 months. Almost all participants exhibited clinical improvement and evidence of myelin repair. In this preliminary study, it was determined that the clinical response to treatment was delayed (2–8 months) and that the intake of 300 mg/day of biotin was the dose associated with the best clinical response [61]. In the MS-SPI randomized controlled trial (RCT) that followed to confirm the efficacy and safety of treatment, high-dose biotin (MD 1003) lowered MS-related disability in 12.6% of progressive MS patients compared to no improvement in the placebo group [5]. However, in the subsequent phase III trial (SPI2), MD 1003 failed to improve disability or walking speed, which formed as the primary composite endpoint, in patients with PPMS. Additionally, treatment failed to improve visual acuity compared to placebo in patients with MS experiencing chronic visual loss [62,63].

The results of the phase III trials were not surprising; although the rationale behind biotin’s neuroprotective effect is interesting, there is a notable lack of preclinical evidence to corroborate its myelin repair potential. Methodological issues in the MS-SPI study may also have resulted in biased results [64,65]. By 2017, Medday Pharmaceuticals had already withdrawn the application for marketing authorization of biotin 100 mg hard capsules for the treatment of progressive MS because of an unfavorable benefit–risk assessment [4].

Treatment with high-dose biotin has also been investigated in several other clinical settings beyond MS. In a pilot study in patients with chronic demyelinating peripheral neuropathies, biotin administration improved various sensory and motor parameters, gait abilities and nerve excitability parameters even though the predefined primary endpoint, which consisted of a composite nerve conduction study criterion was not met [66].

Another pilot study evaluated the safety and efficacy of MD1003 in amyotrophic lateral sclerosis (ALS), a motor neuron disease characterized by progressive death of the upper and lower motor neurons. In this study, treatment with high-dose biotin was safe and well tolerated; however, no significant between-group difference in functional decline was observed. However, this was a small trial in which the two arms were not well balanced during screening; therefore, more research is warranted [67].

The safety, tolerability, and pharmacokinetics of the biotin analog 2-iminobiotin (2-IB) were evaluated in a single-center RCT in patients with ischemic stroke due to large-vessel occlusion of the anterior circulation treated with endovascular thrombectomy. 2-IB is a selective inhibitor of neuronal and inducible nitric oxide synthases (NOS). Nitric oxide has been implicated in the development of reperfusion injury after cerebral hypoxia–ischemia; therefore, it was hypothesized that utilizing 2-IB to inhibit neuronal and inducible NOS would reduce reperfusion-associated tissue injury. Forty patients were randomized to receive 2-IB, or placebo. Treatment with 2-IB was deemed as safe and well tolerated, with fewer serious adverse events than those observed on the placebo arm. The authors noted a lower average plasma concentration of 2-IB in patients treated with intravenous thrombolysis than in those who did not, and lower all-cause mortality in the intervention group than in the placebo group [68].

Biotin supplementation has also been studied in relation to neurodegenerative diseases. Findings from AD research have shown that biotin concentrations may be diminished in the AD brain. In population studies using data from the United Kingdom (UK) Biobank, higher biotin intake was associated with a reduced risk of all-cause dementia, including AD [69,70]. Currently, a research group from Spain is recruiting patients for a clinical trial that aims to evaluate the efficacy of thiamine and biotin supplementation in patients with Huntington’s disease [71].

**Table 1:** Clinical effects of biotin.

Compound	Model/Population	N	Duration	Mechanism of Action	Key Outcomes	Reference
High-dose biotin (100–300 mg/day) (MD1003)	Progressive MS (PPMS/SPMS), pilot study	23	2–36 months	Enhances energy metabolism, activates biotin-dependent carboxylases (myelin repair hypothesis)	Clinical improvement in most patients; delayed response (2–8 months)	[61]
High-dose biotin (oral biotin 100 mg, thrice daily, MD1003)	Progressive MS	642	12 months	Metabolic support, neuroprotection	No significant improvement in disability or walking speed vs. placebo	[62]
High-dose biotin (oral biotin 100 mg, thrice daily, MD1003)	MS-related chronic visual loss	93	6 months	Neuroprotection, myelin repair	No improvement in visual acuity vs. placebo	[63]
High-dose biotin (oral biotin 100 mg, thrice daily)	Demyelinating peripheral neuropathies	15	12 months	Enhances nerve metabolism	Improvements in sensory/motor function and gait; primary endpoint not met	[66]
High-dose biotin (oral biotin 100 mg, three times daily, MD1003)	Amyotrophic lateral sclerosis	30	6–12 months	Mitochondrial/metabolic support	Safe and well tolerated; no significant effect on disease progression	[67]
2-Iminobiotin (biotin analog)	Ischemic stroke patients undergoing thrombectomy	40	Acute (24–48 h)	Inhibits neuronal and inducible NOS → reduces reperfusion injury	Safe, well tolerated; fewer serious adverse events; lower mortality trend	[68]

MD1003: pharmaceutical-grade high-dose biotin formulation; mg: milligrams; MS: multiple sclerosis; NOS: nitric oxide synthase; PPMS: primary progressive multiple sclerosis; SPMS: secondary progressive multiple sclerosis.

## 4. Folic Acid

**Figure 2:**
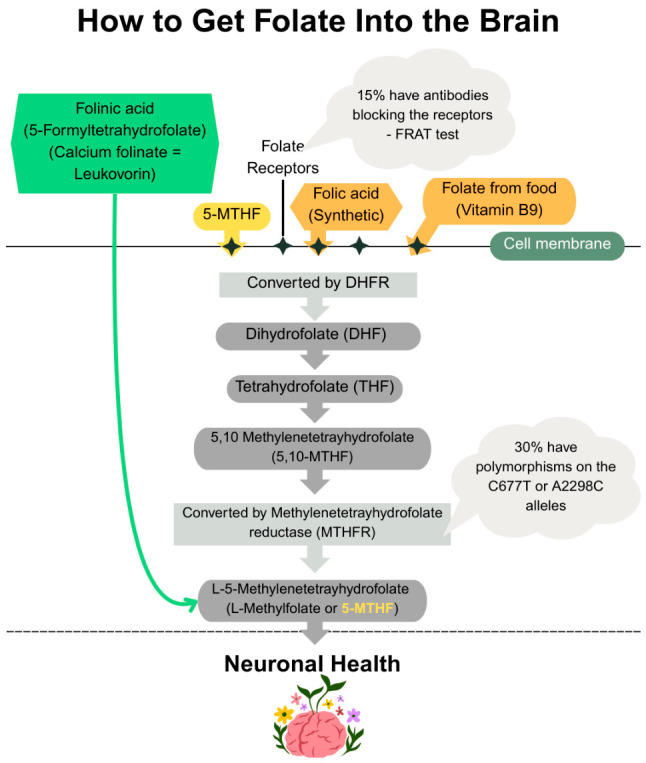
Diagram depicting the steps for the synthesis of methylfolate. Folate, folic acid, and methylfolate all require binding to surface folate receptors in order to enter the brain cells, a process that is prevented in the presence of folate receptor autoantibodies (FRA). In contrast, folinic acid bypasses the folate receptors and the MTHFR, leading to endogenous production of methylfolate even in those with FRA or MTHFR polymorphisms. MTHFR: Methylenetetrahydrofolate reductase.

Folates are a family of compounds composed of pteridine, para-aminobenzoic acid, and glutamic acid. Folate (vitamin B9) can exist as naturally occurring folate, which can be found in leafy greens, vegetables, and legumes, or as the oxidized, much more bioavailable, synthetic folic acid. Tetrahydrofolate (THF) is the active form of folic acid that occurs with the addition of four hydrogen atoms to the 5,6,7, and 8 positions in the pteridine ring (Figure 2) [72]. Usually, THF carries one-carbon units (methyl, methylene, formyl groups) from a donor source, commonly serine, glycine, histidine, or tryptophan, to a biosynthetic intermediate for nucleotide synthesis and methylation reactions. Methylenetetrahydrofolate reductase (MTHFR) catalyzes the conversion of 5,10-methylenetetrahydrofolate to 5-methyltetrahydrofolate (5-MTHF) (Figure 2), which is utilized for the remethylation of homocysteine (Hcy) to methionine which, in turn, is converted to S-adenosylmethionine (SAM) that serves as a methyl group donor for DNA and histone methylation. MTHFR deficiency results in a reduction in SAM and an increase in Hcy concentrations, which in turn is associated with cardiovascular, cerebrovascular, and thromboembolic disease [73,74]. It is estimated that 10% of the population worldwide is homozygous for the *MTHFR* C677T polymorphism, which reduces the activity of the MTHFR enzyme and thus causes high levels of Hcy.

Dysregulation of folate metabolism can result in changes in DNA methylation and increased oxidative stress due to its involvement in the synthesis of glutathione which is an important cellular antioxidant [75]. High levels of Hcy have been associated with alterations in glutamatergic transmission that can affect calcium homeostasis inside the cell, increase ROS production that can cause neuronal damage and cell death, mitochondrial dysfunction through copper (II) chelation, which can result in cytochrome C oxidase inactivation, and inflammatory processes, particularly increased NF-kB expression. However, whether Hcy is the cause or the result of immune activation remains unknown [76].

Folate deficiency may cause macrocytic anemia, glossitis and atypical neuropsychiatric symptoms, such as irritability, cognitive impairment, and mood changes [77]. Folic acid is widely used in supplements and fortified foods to reduce the risk of neural tube defects (NTDs) associated with maternal folate deficiency. While debate continues over universal mandatory fortification, its critical role during the periconceptional period in supporting fetal central nervous system development underpins arguments for fortification. NTDs, including anencephaly, spina bifida, and encephalocele, affect roughly 1 in 1000 pregnancies in Europe [78]. Guidelines regarding periconceptional supplementation recommend that all women trying to conceive should take 400 mcg of folic acid daily to reduce the risk of NTDs [79,80]. Pregnant women at a greater risk of being affected by an NTD should receive 4000 mcg of folic acid daily, starting one month pre-conception and continuing supplementation throughout the first trimester of gestation [81]. Folic acid fortification is associated with a lower prevalence of NTDs and eliminates individual initiative and barriers to consistent use and promotes equality across socioeconomic groups. A recent systematic review and meta-analysis demonstrated a 44% reduction in NTD risk associated with fortification [82].

The argument against global fortification is the concern that fortification will need to expose thousands of people to folic acid for each case of NTD prevented. A major concern regarding folic acid supplementation is that increased intake of folic acid could mask the anemia caused by B12 deficiency in the elderly and increase the prevalence of neurological disease. In countries where mandatory fortification is implemented, elderly with high folate and low-to-normal vitamin B12 concentrations have been shown to exhibit cognitive impairment. In addition, high levels of folate and low B12 during pregnancy have been associated with an increased risk of insulin resistance and obesity in the offspring [83].

The range of benefits associated with folic acid is constantly being updated. There is interest in its anticancer properties, protective effects against cardiovascular disease, diabetes, and obstetric outcomes. The effect of folic acid in neurocognitive disorders has also been studied; there are numerous studies suggesting a protective effect against the risk of stroke and other neurocognitive disorders [84].

### 4.1. Preclinical Studies

In the Lipopolysaccharide (LPS)-injured model of neuroinflammation, Darbandi et al. showed that folic acid supplementation restored the elevated oxidative stress markers and reduced the pro-inflammatory cytokines associated with LPS in rats [85].

There is growing evidence to suggest that high Hcy levels are related to the progression of AD. The mechanisms involved result in endothelial dysfunction and neuronal damage and obstruction of the proliferation of neural stem cells via the DNA methylation pathway. As previously mentioned, SAM is the main methyl donor for DNA and histone methylation. SAM is also involved in DNA methylation processes that regulate the expression of genes involved in Aβ production, including the amyloid precursor protein (*APP*) and presenilin 1 (*PS1*) genes [86]. Lin et al. showed that hypomethylation of *PS1* gene promoters increases Aβ production in brain 2 (BV-2) cells [86,87]. Kruman and associates showed that elevated Hcy paired with low folic acid concentrations caused Aβ-induced neuronal death in hippocampal cultures and promoted neuronal degeneration in APP mutant transgenic mice by the accumulation of DNA damage and impaired DNA repair [88].

Tau hyperphosphorylation consists of a hallmark of AD, propelling the formation of neurofibrillary tangles. Tau dephosphorylation is catalyzed by protein phosphatase 2A (PP2A); therefore, downregulation of the PP2A promoter methylation accelerates tau phosphorylation in the AD brain. Sun et al. showed that Hcy was able to induce AD-like changes in the hippocampus and that folic acid and SAM significantly reduced the Hcy -induced AD changes, including tau protein phosphorylation and Aβ expression. Hcy also increased PS1 expression and decreased PP2A activity, whereas the administration of folic acid and SAM was able to ameliorate this effect by modulation of PS1 and PP2A methylation levels [86].

In healthy rats, short-term memory, long-term memory and motor coordination improve with folic acid supplementation [89]. Further studies in PD models have shown that folic acid could be used to ameliorate motor symptoms as well. In a MPTP-induced mouse model of PD, supplementation with folic acid improved motor performance and reduced levels of Hcy and oxidative damage in the substantia nigra [90]. However, in a 6-hydroxydopamin (6-OHDA)-induced PD model, supplementation with folic acid attenuated the severity of PD symptoms; however, Hcy levels were significantly greater in the high-intake-of-folic-acid group than in the control group [91].

The effect of folic acid and Hcy on brain ischemia models was also studied. According to Gou and associates Hcy inhibits hippocampal neurogenesis in the focal ischemic rat brain by inhibiting DNA methylation [92]. Excessive N-methyl-d-aspartate receptor (NMDAR) activation triggers synaptic dysfunction and excitotoxic neuronal death in ischemic brains among other neuropsychiatric and neurological disorders. In ischemic stroke models, Liang et al. showed that folic acid supplementation reduces neuronal death and improves synaptic dysfunction by downregulating NMDAR expression [93].

In a preclinical study, a model of depression was established through chronic unpredictable mild stress in rats. The effect of folic acid supplementation was studied, along with levels of Hcy, monoamine neurotransmitters, IL-6, BDNF, and β-endorphin levels. Zhou and associates reported an antidepressant effect of folic acid and increased monoamine neurotransmitters, BDNF and β-endorphin, and concurrently reduced levels of IL-6 and Hcy [94]. Similar anti-depressive results have also been recorded in other murine models [95,96]. Table 2 summarizes the mechanisms and preclinical and clinical effects of folic acid on brain health.

### 4.2. Clinical Studies

As described above, studies have shown that elevated Hcy consists of a risk factor for the development of neurodegenerative disease, including PD. PD is characterized by a selective loss of dopaminergic neurons in the substantia nigra pars compacta that results in clinically evident rigidity, resting tremor and bradykinesia. The pathophysiological mechanisms of the disease involve the ubiquitin–proteasome system, dysregulation of mitochondrial function and oxidative stress. Dopamine precursors (levodopa) and other dopamine agonists and catechol-O-methyltransferase (COMT) inhibitors have been used for treatment.

High Hcy has been associated with the pathogenesis and progression of the disease by accelerating loss of dopaminergic neurons. It has also been associated with depression and cognitive impairment in patients with PD [97,101]. The etiology of hyperhomocysteinemia is diverse; one of the possible causes would be folate deficiency but in prospective studies, an association between folate intake and PD has not been established [102,103]. However, a recent metanalysis of 24 studies showed that PD patients have lower plasma levels of folic acid than healthy controls. This reached statistical significance in patients with PD and cognitive impairment [104].

In animal and human studies, treating PD with levodopa has caused elevations in Hcy plasma concentrations. In this case, the increase in Hcy is the result of the O-methylation of L-dopa by COMT, which utilizes SAM as a methyl donor, propelling the production of Hcy. Combination of levodopa with a COMT inhibitor reduces the increase in Hcy levels [105,106]. The hypothesis that co-administration of B vitamins to reduce Hcy levels in levodopa-treated patients has been studied. In a preliminary study, supplementation with B vitamins lowered plasma Hcy below the cardiovascular risk level [106]. The lowering effect of B12 and folate on Hcy levels in patients treated with levodopa for PD has been verified in other studies [107,108].

The effect of folic acid on Hcy levels, inflammation and amyloidogenesis has also been explored in patients with mild cognitive impairment, dementia and Alzheimer’s disease. Ma et al. conducted a series of studies investigating the effect of folic acid supplementation on various inflammatory markers, metabolites of the methionine cycle, amyloid beta peptides and cognitive function tests in patients with mild cognitive impairment. Supplementation with 400 μg/day of folic acid improved levels of Hcy, SAM, Aβ-42, peripheral IL-6, TNF-α and improved cognitive function in patients with mild cognitive impairment. The combination of folic acid and vitamin B12 supplementation was superior to folic acid or vitamin B12 alone [98,109,110,111].

A systematic review and meta-analysis of seven studies also showed that folic acid supplementation had a positive impact on cognitive function in older adults with mild cognitive impairment (MCI) and reduced most inflammatory cytokines, Hcy and biomarkers of AD [99]. Another systematic review and meta-analysis also supported the supplementation of folic acid for improvement of cognitive function and concluded that supplementation with more than 3 mg folic acid improved cognitive function in AD patients [112].

The effect of folic acid supplementation on cognitive function also depends on the local folic acid supplementation policy. In a systematic review and meta-analysis, Zhang and associates showed that folate-based B vitamin supplementation improved cognitive function in older adults in countries without mandatory food folic acid fortification, but no effect was noted in geographical areas with mandatory fortification [113].

Throughout the years there are numerous studies that defend the effectiveness of folate in stroke prevention and recovery. The apparent mechanism would be that low folate results in elevated levels of Hcy, which is a well-documented risk factor for cardiovascular disease, including stroke [76,114]. In a systematic review and meta-analysis that included 12 RCTs, folic acid reduced the risk of stroke by 10% [115]. Folate and Hcy levels are also associated with recovery outcomes after a stroke. A recent study that assessed folate and Hcy in patients with anterior ischemic stroke caused by large vessel occlusion who underwent successful recanalization, showed that lower levels of folate and higher serum Hcy on admission were independently associated with the 90-day futile recanalization risk, a phenomenon where successful recanalization fails to translate into functional independence [100].

Folate also assists in the formation of tetrahydro-biopterin (BH4), which activates tyrosine and tryptophan hydroxylases for the synthesis of tri-mono-amines serotonin, norepinephrine and dopamine [116]. Low levels of monoamine neurotransmitters have been implicated in the pathogenesis of depression [75]. In a systematic review and meta-analysis that was published in 2017, the gathered evidence did not support the addition of folic acid to ongoing antidepressant therapy [117]. However, in an umbrella meta-analysis on the effect of folate supplementation as an add-on for relieving depressive symptoms, Gao et al., concluded that folate supplementation significantly relieved symptoms of depression [118]. Clinician guidelines for the treatment of psychiatric disorders with nutraceuticals and phytoceuticals by the World Federation of Societies of Biological Psychiatry and the Canadian Network for Mood and Anxiety Treatments task force provided up-to-date, evidence-based guidelines for the appropriate use of nutraceuticals and phytoceuticals for the treatment of psychiatric disorders. In these guidelines, they recommended the use of methylfolate for adjunctive use in major depressive disorder and for schizophrenia [119].

Not all forms of vitamin B9 are equal. The vitamin exists in several distinct chemical forms—each with unique properties, bioavailability, and how they are metabolized in the body: (a) Dietary folate is naturally occurring in foods such as leafy greens and legumes; (b) Folic acid is the synthetic form used in fortified foods and many supplements; (c) 5-MTHF is the biologically active methylated form. (d) Folinic acid (Calcium folinate/Leucovorin) is a natural form that is utilized more efficiently. In particular, folate must enter the cells by binding to specialized surface receptors (Figure 2). About 2–3% of apparently healthy children and up to 15% of adults have FRA. However, in certain populations like children with autism spectrum disorder (ASD) or cerebral folate deficiency, the percentage of individuals with FRA can be as high as 60% [120,121,122]. As previously mentioned, a significant portion of the population harbors *MTHFR* gene polymorphisms, with estimates suggesting 60–70% of the population having at least one common variant (*C677T* or *A1298C*). For the most common *C677T* variant, roughly 40% of Caucasians and Hispanics are carriers (heterozygous), and 8–20% are homozygous for two copies, severely impacting enzyme function [123,124,125,126].

Because it bypasses the need for enzymatic conversion by MTHFR, 5-MTHF is especially beneficial for individuals who may carry *MTHFR* variants that impair folate metabolism. Instead, folinic acid (5-forml tetrahydrofolate, calcium folinate, Leucovorin) bypasses the surface folate receptors and enters the cells via a pump. Moreover, folinic acid enters cellular folate pools without needing initial conversion by MTHFR, unlike folic acid (Figure 2). In other words, folinic acid is the only form to support those with folate receptor antibodies and MTHFR polymorphisms. A number studies have shown that the use of Calcium folinate has substantially improved language development in children with ASD [127,128,129].

There are liquid dietary supplements such as VitalFolinic with 5-MTHF™ that are specifically engineered to address common metabolic and transport challenges associated with standard synthetic folic acid supplementation [130].

## 5. Palmitoylethanolamide (PEA)

### 5.1. Introduction

PEA is an endocannabinoid-like lipid mediator that belongs to the N-acyl-ethanolamine fatty acid amide family and acts on multiple molecular targets to exert antimicrobial, anti-inflammatory, analgesic, immunomodulatory, and neuroprotective effects. Its various targets include primarily the Peroxisome proliferator-activated receptor alpha (PPAR-α), as well as the novel cannabinoid receptor G protein-coupled receptor 55 (GPR55) and G protein-coupled receptor 119 (GPR119). PEA can also downregulate mast cell activation, a mechanism described as autacoid local injury antagonism. Its ability to indirectly activate cannabinoid receptors 1 and 2 (CB1 and CB2) by inhibiting the degradation of anandamide is known as the entourage effect. Through the entourage effect, PPAR-α activation and its action as an allosteric modulator, PEA can activate and desensitize the transient receptor potential vanilloid receptor 1 channels to exert an anti-nociceptive effect. Finally, PEA has been reported to reduce adipose cell leptin release via lipopolysaccharide (LPS)-stimulated pathways, an effect that possibly contributes to its anti-inflammatory activity [131,132,133].

PEA was first identified in chicken egg yolk as an active anti-inflammatory agent in the 1950s [134]. The discovery of the endocannabinoid system rekindled interest in PEA, and over the past two decades, there has been increased interest in the development and use of PEA-containing formulations for chronic pain, inflammation, and certain brain diseases [134].

Endogenously, PEA is synthesized from palmitic acid “on demand” as a protective response to cellular injury and is therefore upregulated during disease. The first step in its synthesis is the Calcium- and cyclic adenosine monophosphate (cAMP)-dependent transfer of palmitic acid from phosphatidylcholine to phosphatidylethanolamine to form N-acylphosphatidylethanolamine (NAPE), which is subsequently cleaved by a NAPE-specific phospholipase D to release PEA. PEA is inactivated via fatty acid amide amidohydrolase (FAAH) or PEA-preferring acid amidase to form palmitic acid and ethanolamine [134].

The exogenous administration of PEA presents certain challenges; its lipophilic nature and poor solubility and bioavailability have complicated its development as a nutraceutical. PEA can cross the blood–brain barrier after oral administration; however, only in small amounts. Strategies to improve the absorption of lipid-based supplements, such as PEA, include emulsification, micronized dispersion, or utilizing specialized delivery systems designed to increase the dispersion of lipophilic agents in aqueous environments [133].

### 5.2. Preclinical Studies

One strategy to mitigate the neurological sequelae of AD is to target neuroinflammation which, begins early in the natural course of AD, as glial cells respond to the abnormal accumulation of proteins. Scuderi et al. conducted an in vitro study that demonstrated the ability of PEA to reduce Aβ-induced astrocyte activation and pro-inflammatory molecule and cytokine release in primary rat astrocytes through a PPAR-dependent mechanism [134,135,136]. The same arm subsequently demonstrated the protective effects of PEA against Aβ-induced toxicity in primary rat mixed neuroglial co-cultures and organotypic hippocampal slices [137,138]. Indeed, PEA has shown anti-inflammatory and neuroprotective properties in several other preclinical models of AD [139,140,141,142]. In another study, co-ultramicronized PEA and the antioxidant luteolin were able to counteract the reduction in glial cell line-derived neurotrophic factor (GDNF) and BDNF mRNA levels in addition to Aβ-induced neuroinflammation and glial activation in an AD rat model [143].

The effects of PEA on cerebral ischemia have also been studied. Richter et al. evaluated whether PEA affected cortical spreading depression (CSD), a depolarization wave in the cerebral gray matter that occurs after traumatic brain injury (TBI) or cerebral ischemia and deteriorates brain damage. They elicited and evaluated CSD before and after intraperitoneal PEA injection in adult rats. PEA stabilized CSD amplitudes for at least four hours, an effect attributed to the inhibition of pro-inflammatory cytokine release [144]. In another rat model that underwent middle cerebral artery occlusion, the effects of a co-ultramicronized combination of PEA and luteolin were studied. Caltagirone and associates showed that rats treated with PEA had reduced edema and brain infarct volume, improved neurological scores, reduced pro-inflammatory and astrocyte markers, and restored expression of GDNF and BDNF. The same authors also conducted an observational human study in which co-ultraPEALut (Glialia^®^) was administered during rehabilitative therapy after a stroke. After treatment, patients had statistically significant improvements in their neurological status, cognitive abilities, pain, degree of spasticity, and independence in daily living activities [145].

Fewer preclinical studies have examined the effects of PEA on PD models. Previous studies have shown that PPAR-α is expressed by dopamine neurons of the substantia nigra and spiny neurons of the dorsal striatum; therefore, it could be a therapeutic target for neurodegenerative disorders, including PD. Fenofibrate which is a selective PPAR-α agonist, prevented dopaminergic neuronal loss in the neurotoxin 1-methyl-4-phenyl-1,2,3,6-tetrahydropyridine (MPTP) mouse model of PD. However, bezafibrate, another synthetic PPAR-α agonist, had no effect in the same study [146]. Esposito et al. used PPAR-αKO and PPAR-αWT mice to assess the effect of PEA against minocycline in the MPTP model of PD. PEA reduced dopaminergic cell death and glial activation and mitigated motor deficits. Compared to minocycline, PEA demonstrated superior recovery in PEA-treated mice. Interestingly, PPAR-αKO mice exhibited exacerbated toxicity from MPTP, indicating that PEA neuroprotection was dependent on PPAR-α [147].

In another mouse model of PD induced by the striatal injection of 6-OHDA, PEA improved behavioral impairments by reducing several pathogenic factors at the striatal level. Similar protective effects of PEA against neuronal damage by 6-OHDA were also observed in SH-SY5Y neuroblastoma cells [148].

### 5.3. Clinical Studies

PEA has been meticulously studied for its analgesic effects on musculoskeletal disorders, neuropathic pain, chronic pain, and dysmenorrhea [149]. However, clinical studies evaluating its effects on brain diseases are limited. This could be associated with the complexity of the pharmacokinetics and bioavailability of PEA. New PEA formulations subjected to ultramicronization will improve the bioavailability of the compound and allow more clinical studies to be conducted. Indeed, most high-quality clinical studies were fairly recent, and several are currently ongoing.

A review of the literature revealed relatively few studies evaluating the effectiveness of PEA in patients with AD, mild cognitive dysfunction, and dementia. Therefore, the recent phase 2 study by Assogna and associates investigating the effect of PEA supplementation combined with luteoline in patients with frontotemporal dementia (FTD) is of particular interest. FTD is a devastating disease associated with synaptic dysfunction, neurotransmitter deficits, and neuroinflammation, and is clinically characterized by a rapid decline in global functioning and reduced life expectancy; however, to date, no effective pharmacological treatment is available. In addition to its anti-inflammatory effects, PEA can modulate synaptic activity by enhancing GABAergic (gamma-aminobutyric acid) neurotransmission through the activation of the CB1 receptor at a presynaptic site. Hence, investigating its efficacy and safety in patients with FTD is warranted.

This was a phase 2, monocentric, randomized, double-blind, placebo-controlled trial. Forty-eight participants were randomized to receive co-ultraPEAlut oral suspension or placebo for 24 weeks. The primary efficacy outcome measure was the change from baseline in the Clinical Dementia Rating (CDR), Dementia Staging Instrument and another scale specific to patients with FTD. Patients in the intervention group showed less decline in global disease severity and functional decline compared with those treated with a placebo. No significant effect of co-ultraPEAlut treatment on frontal executive function or behavioral symptoms was found [150].

The ability of the co-ultramicronized combination of PEA and luteolin supplementation to restore BDNF expression [145] was also investigated in clinical trials. A randomized, double-blind, placebo-controlled crossover study based in London investigated the effects of PEA supplementation on cognitive health and serum BDNF concentrations. Thirty-nine healthy university students were randomized to take PEA in a formulated form known as Levagen+ (Gencor Pacific Limited, Lantau Island, HK) or a placebo. Following six weeks of PEA supplementation, serum BDNF expression significantly increased, and there was a significant improvement in memory-related outcomes. No significant differences were observed in attention, psychomotor speed, and executive function parameters [151].

A small, very recent RCT by the same group explored whether Levagen+ had stress-modulating effects. In this study, 16 female university students were randomly assigned to receive PEA or a placebo for 6 weeks. Stress was assessed by recording heart rate variability, subjective stress, mood measures, and salivary cortisol concentrations. PEA supplementation had a positive effect on markers associated with enhanced physiological stress regulation; however, subjective stress measures and salivary cortisol concentrations remained unchanged [152].

The same PEA formulation has previously been evaluated for its effect in patients with sleep disturbances, where PEA significantly reduced the amount of time required to fall asleep in patients with sleep latency issues [153]. It also improved cognition upon waking and the time needed to feel fully awake. Fast sleep and better awakening are both desired attributes that are currently lacking in approved pharmaceutical options for sleep disturbances [153].

Another study investigated whether PEA supplementation shortly after ischemic stroke could improve patient outcomes following thrombolysis. Sixty patients who had an ischemic stroke were randomized to receive either thrombolytic therapy (alteplase) alone or in combination with an oral suspension formulation that contained PEA and luteolin called PEALut (GlialiaR, Epitech Group SpA, Saccolongo, Padova, Italy) within 72 h after the ischemic event. Neurological deficits, independence in activities of daily living and cognitive impairment were assessed at baseline, at discharge from the Stroke Unit and after 90 days. Patient independence and mobility in daily living activities showed a significant increase in the PEALut group compared to the control group. Recovery of cognitive function was also greater in the intervention group than in the thrombolysis-alone group [154]. A study that will evaluate the effect of co-ultramicronized PEA and luteolin on the clinical outcomes of patients with acute ischemic stroke undergoing mechanical thrombectomy is underway [NCT06777680] [155].

The effects of PEA have also been investigated in neuropsychiatric disorders. Neuroinflammatory pathways, dysregulation of glutamate transmission, and deficits in the endocannabinoid system are all involved in the pathogenesis of major depressive disorder and mood disorders [156]. In adults with major depressive disorder (MDD), 600 mg twice daily of ultra-micronized PEA (ACER, Tehran, Iran) as adjunctive therapy to citalopram improved scores in the Hamilton Depression Rating Scale, demonstrated significantly greater improvement in depressive symptoms and higher response rates at the end of the trial [156]. In a small RCT, the same dose and formulation of PEA improved manic symptoms and overall clinical status when given as an adjunct on treatment with lithium and risperidone in patients in the acute phase of mania [157]. Finally, 600 mg of PEA twice daily, alongside risperidone, showed greater symptom improvement in patients with primary negative schizophrenia [158]. Table 3 summarizes the clinical evidence on PEA.

### 5.4. Clinical Studies of PEA on Pain

PEA has been extensively investigated in clinical settings primarily for its analgesic and anti-inflammatory properties, with the strongest evidence emerging from studies on chronic pain, neuropathic pain, and gynecological pain disorders (Table 4). Multiple randomized and controlled trials have demonstrated that PEA, administered typically at doses ranging from 600 to 1200 mg/day, is effective in reducing pain and improving functional outcomes in conditions such as knee osteoarthritis, diabetic peripheral neuropathy, carpal tunnel syndrome, and temporomandibular joint disorders [159,160,161,162,163]. In these populations, PEA consistently reduced pain intensity and, in some cases, improved nerve function and quality of life. However, not all findings are uniformly positive; for example, a randomized, double-blind, placebo-controlled trial in patients with spinal cord injury-related neuropathic pain did not demonstrate a significant benefit over placebo [164], highlighting potential variability depending on disease etiology and severity.

PEA has also shown efficacy in visceral and pelvic pain conditions, particularly when combined with polydatin. Clinical studies in patients with irritable bowel syndrome (IBS) [165] and endometriosis-associated pelvic pain [166] reported reductions in symptom severity and pain scores following treatment with combined formulations. Similarly, benefits have been observed in primary dysmenorrhea and vestibulodynia [167,168], supporting a role for PEA in modulating inflammation-driven and hormonally influenced pain pathways.

**Figure 3:**
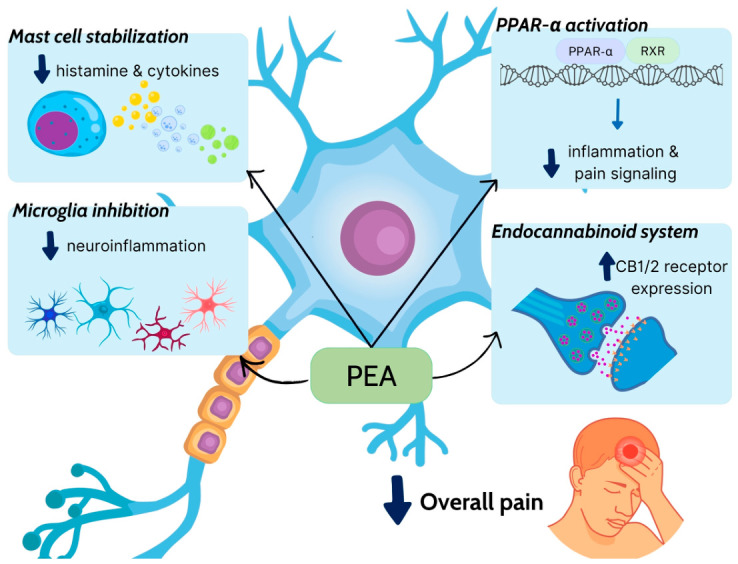
Schematic representation of mechanisms of action of PEA in pain modulation. CB1: cannabinoid receptor type 1; CB2: cannabinoid receptor type 2; PEA: palmitoylethanolamide; PPAR-α: peroxisome proliferator-activated receptor alpha; RXR: retinoid X receptor.

Mechanistically, the clinical effects of PEA are largely attributed to its ability to activate PPAR-α, modulate mast cell activity, and regulate neuroinflammation through interactions with the endocannabinoid system and transient receptor potential channels (Figure 3). Notably, the bioavailability of PEA appears to be formulation-dependent, with ultramicronized and co-formulated preparations showing improved clinical efficacy in several studies. Overall, current clinical evidence supports the use of PEA as a safe and moderately effective therapeutic option for a range of inflammatory and neuropathic pain conditions, although further large-scale and standardized trials are needed to better define its efficacy across different patient populations and dosing strategies.

**Table 4:** Clinical effects of Palmitoylethanolamide (PEA) on pain.

Compound	Model/Population	N	Dosage	Duration	Mechanism of Action	Key Outcomes	Reference
PEA + Polydatin	Patients with irritable bowel syndrome (IBS)	54	PEA 400 mg + Polydatin 40 mg, BID	12 wks	Anti-inflammatory, mast cell modulation, gut–brain axis regulation	↓ abdominal pain sensation, improved IBS symptoms	[165]
PEA	Patients with knee osteoarthritis	74	300–600 mg/day	8 wks	Anti-inflammatory, analgesic (PPAR-α activation)	↓ pain sensation, improved function	[159]
PEA (ultramicronized)	Spinal cord injury neuropathic pain	73	600 mg BID	12 wks	Neuromodulatory, anti-inflammatory	No significant benefit vs. placebo	[164]
PEA	Relapsing–remitting multiple sclerosis	29	600 mg/day	6 months	Anti-inflammatory, cytokine modulation	↓ IFN-β side effects, ↓ cytokines	[160]
Micronized PEA + Transpolydatin	Endometriosis-related pelvic pain	47	PEA 400 mg + Polydatin 40 mg, BID	3 months	Anti-inflammatory, analgesic	↓ pelvic pain sensation	[166]
PEA	Carpal tunnel syndrome	61	600 mg/day	60 days	Neuroprotective, anti-inflammatory	↓ pain, improved nerve function	[161]
PEA	TMJ inflammatory pain	24	300 mg BID	14 days	Anti-inflammatory, analgesic	Pain relief comparable/superior to NSAIDs	[162]
PEA + Polydatin + TENS	Vestibulodynia	20	PEA 400 mg + Polydatin 40 mg, BID	60 days	Anti-inflammatory, neuromodulatory	↓ vulvar pain sensation, improved function	[168]
PEA (ultramicronized)	Burning mouth syndrome	41	600 mg BID	8 wks	Neuromodulatory, anti-inflammatory	↓ burning pain sensation	[169]
PEA	Diabetic peripheral neuropathic pain	70	600 mg BID	8 wks	Neuroprotective, anti-inflammatory	↓ neuropathic pain sensation	[163]
PEA + Polydatin	Primary dysmenorrhea	56	PEA 400 mg + Polydatin 40 mg, BID	3 months	Anti-inflammatory, analgesic	↓ menstrual pain sensation	[167]

BID: twice daily; IBS: irritable bowel syndrome; IFN-β: interferon beta; mg: milligrams; NSAIDs: nonsteroidal anti-inflammatory drugs; PEA: palmitoylethanolamide; PPAR-α: peroxisome proliferator-activated receptor alpha; TMJ: temporomandibular joint; TENS: transcutaneous electrical nerve stimulation; wks: weeks; ↓: decreased.

## 6. Huperzine A

### 6.1. Introduction

In Chinese medicine, *Huperzia serrata* (toothed clubmoss) is traditionally used to treat fever and inflammation, certain blood disorders, and even schizophrenia. Its medicinal properties are mostly attributed to alkaloids, such as lycodoline, lycoclavine, serratinine, and huperzines. This plant, which requires several years to mature, is currently at risk of extinction; therefore, developing methods to synthesize some of its compounds, particularly huperzines, has gained interest. *Huperzine A* (HupA) is a sesquiterpene alkaloid that comprises less than 0.02% of *H. serrata*. Its skeleton contains an ethylidene group and an aromatic pyridone moiety fused with a bicyclic ring system bearing a primary amino group [170].

In recent years, considerable research has been conducted on this phytochemical compound to elucidate its therapeutic effects, chemical synthesis, pharmacokinetics, and to evaluate possible toxicities. HupA is an attractive compound because it is a potent acetylcholinesterase (AChE) inhibitor and NMDAR antagonist [171]. AChE is available in the CNS of adults as a globular tetramer (G4 AchE) and HupA exhibits the best selectivity and efficacy against tetrameric AchE [172]. Cholinesterase inhibitors increase acetylcholine (ACh) at the neuronal synaptic cleft by inhibiting the enzyme responsible for the hydrolysis of ACh resulting in improved neuronal transmission. HupA inhibits AChE through a non-covalent, reversible interaction with aromatic amino acids located in the catalytic gorge. The HupA–AChE complex has a low dissociation rate, which makes HupA a slow, reversible inhibitor [173].

### 6.2. Preclinical Studies

*In vivo* studies have shown that HupA has antioxidant properties and can inhibit PKC/MAPK, γ-secretases and increase phospho-glycogen synthase kinase-3 (GSK-3) in transgenic mice resulting in enhanced learning capacity and memory [174]. HupA has also exhibited anti-inflammatory potential by lowering pro-inflammatory cytokines and enhancing anti-inflammatory cytokine production in neuroinflammation. In a mouse model of MS, it ameliorated disease severity by reducing the accumulation of inflammasomes and decreasing the neuronal injury [175]. In several animal models, HupA also potentiates GABA signaling and suppresses seizures [173]. HupA restores lost GABA-mediated inhibition after TBI in a TBI rat model which can clinically be used as an anticonvulsant and antinociceptive [176]. Mei et al. also showed that HupA can decrease neuroinflammation in a repetitive brain injury model [177].

In aging rats receiving D-galactose to induce severe blood–brain barrier dysfunction and to decrease the density of tight junctions and induce apoptosis in the hippocampus, subcutaneous administration of HupA for 8 weeks inhibited AChE activity, reduced neurovascular damage by inhibiting NF-κB activation and improved cerebrovascular function by suppressing the decrease in the density of tight junctions and cell apoptosis [178]. In addition to inhibiting acetylcholine activity, intraperitoneal administration of HupA suppressed activation of glial cells in the ischemic penumbra, decreased overexpression of pro-inflammatory factors and inhibited nuclear translocation of NF-κB in a rat model of transient focal cerebral ischemia. These actions resulted in significant restoration of regional cerebral blood flow, reduction in infarct size, and reduction in neurological deficit score 24 h after reperfusion. Similar beneficial effects were observed after HupA administration for 14 days [179].

Despite the amount of positive feedback from preclinical trials, there is evidence that there are critical inter-species differences, for example, in the cholinesterase activity in humans and rodents, that need to be taken into account before applying the effects of HupA in animal models to humans [170].

### 6.3. Clinical Studies

Older studies have revealed that HupA is safe for patients with AD and effective in improving memory and behavior in this cohort [180,181,182,183].

Cholinergic degeneration is associated with cognitive deficits in AD; therefore, enhancing cholinergic neurotransmission using AChE inhibitors has been the hallmark of AD treatment. Tacrine, donepezil, rivastigmine, and galantamine have proven useful in alleviating some symptoms; however, none have been effective in preventing disease progression. A particular concern with AChE inhibitors is the number of adverse effects related to the activation of peripheral cholinergic systems. Gastrointestinal symptoms (diarrhea, nausea, and vomiting) and cardiovascular effects (syncope, bradycardia, and heart block), along with neuropsychiatric (tremor, insomnia, and seizures), are common. The first-generation AChE inhibitor tacrine has been discontinued due to severe hepatotoxicity and low efficacy [184]. HupA has the dual advantage that it acts as a selective AChE inhibitor and a potent NMDA receptor antagonist; these attributes make HupA a useful agent for both the prevention of the pathogenesis of the disease and symptom control [170].

In 2013, Yange et al. performed a systematic review and meta-analysis of RCTs to determine the effectiveness of HupA in patients with AD. Although the authors determined that there was a high risk of bias in the 20 included RCTs, HupA improved cognitive function, daily living activity, and global clinical assessment compared to no treatment, psychotherapy, and conventional medicine. The differences in the measurement of each outcome, duration of treatment, reporting methods and publication bias were limitations of this systematic review [185]. Later, in a preliminary trial that randomized 110 patients with AD to receive placebo, rivastigmine, galantamine, and HupA for 24 weeks in combination with memantine, Shao et al. showed that the addition of 100 μg twice daily of HupA to memantine resulted in better MMSE and activities of daily living scores than the addition of a placebo [186].

A more recent double-blind study evaluated the effects of HupA on cognitive function and task switching in patients with AD and healthy individuals. Task switching is a higher-order executive function that relies on the frontoparietal network and is affected in patients with AD. Post-treatment scores on the Addenbrooke’s Cognitive Examination (ACE) and Trail making test (TMT), which assess visual attention and task switching, were significantly improved after 8 weeks of treatment with HupA [187]. In another recent parallel-group randomized controlled trial, administration of intramuscular HupA postoperatively in patients that underwent neurosurgery under general anesthesia, improved significantly mini mental state examination (MMSE) scores over the first four postoperative days [188].

A multicenter, double-blind, double-dummy, active- and placebo-controlled, parallel-group RCT that will initially evaluate the efficacy and safety of different doses of HupA controlled-release tablets in patients with mild-to-moderate Alzheimer’s type dementia is underway. At the efficacy confirmation stage, this study aims to assess the effect of HupA controlled-release tablets on cognitive function and functional abilities in these patients, and in the open-label extension stage, further evaluate the long-term efficacy and safety of the treatment [NCT07066826] [48].

A systematic review and meta-analysis of randomized trials conducted by Dang et al. evaluated the pharmacological treatments available for vascular dementia. In this review, 16 RCTs were identified on HupA, including 572 patients and 542 controls. HupA showed superior efficacy to donepezil and was among the most effective drugs in terms of changes in the MMSE and activities of daily living scores; however, it had the poorest safety profile [189].

Zafonte and associates conducted a single-site, randomized, double-blind, placebo-controlled Phase II study, to determine the effect of HupA treatment for 12 weeks on memory function in patients with moderate to severe TBI. For neurocognitive evaluation, the California Verbal Learning Test-II (CVLT-II) was utilized to assess verbal learning and memory. CVLT-II total learning, short delay free recall, and long delay free recall were assessed. Owing to the small number of participants who completed the 12-week treatment (n = 12), the authors used permutation testing to increase the power of this study. After 12 weeks of intervention, there was no difference between the two groups in memory performance, self-reported depression measured by the Beck Depression Inventory (BDI) test, and seizure activity in patients with subacute to chronic moderate to severe TBI. Surprisingly, the placebo arm showed notable improvement in all three memory batteries, as well as in the BDI scores. No significant between-group difference in the prevalence of adverse effects was observed. This study could illuminate a unique responsiveness to placebo in this patient cohort; however, given the small sample size, this was an underpowered study and could not confidently determine the effect of HupA treatment in patients with TBI [190].

Studies have shown changes in the cholinergic system in schizophrenia, resulting in cognitive deficits at a very early stage. A systematic review and meta-analysis showed that patients with schizophrenia have a decrease in muscarinic and nicotinic receptor levels, particularly in M1/M4 muscarinic receptors in the striatum, hippocampus and fronto-cingulate cortex [191]. AChE inhibitors have been previously applied for improving cognitive impairment in people with schizophrenia, therefore HupA could also be beneficial for cognitive function. Due to conflicting results, Zheng et al. conducted a systematic review and meta-analysis of randomized controlled trials to determine the efficacy of HupA for cognitive deficits in schizophrenia spectrum disorders [192]. The primary outcome was the cognitive effect evaluated by any battery and the secondary outcomes included improvement in psychiatric symptoms. Twelve Chinese RCTs were included in the synthesis; however, they were deemed as being of low quality due to the lack of double-blind design and high risk of selection bias. Cognitive function and psychiatric symptoms improved in the HupA arm; however, there was no difference in the acute phase of illness. No significant differences were detected between the two arms regarding common side effects (weight gain, liver enzyme elevation, electrocardiographic abnormality, nausea/vomiting, insomnia, constipation, excitement/agitation, muscle rigidity, or tremor) [192].

Another systematic review of RCTs investigated the efficacy and safety of HupA administration in patients with major depressive disorder; however, only three low-quality trials from China were identified, with an average intervention duration of 6.7 weeks. Therefore, no definitive conclusions could be made regarding the effect of HupA in patients with major depressive disorder [193].

## 7. *Hericium erinaceus* (Lion’s Mane)

### 7.1. Introduction

Mushrooms have long been valued for their nutritional and therapeutic applications in East Asia. There has been considerable interest in identifying and utilizing bioactive compounds found in the mycelium or fruiting body of certain mushrooms for the prevention and treatment of diseases. The most important bioactive compounds are the polysaccharides of the fungal cell wall, α- and β-glucans, which are well known for their antitumor, immunomodulatory, antioxidant, anti-inflammatory, antimicrobial, and antidiabetic activities [194].

*Hericium erinaceus* (HE), an agaricomycete, is a core ingredient in several nutraceutical products for cognitive enhancement and gastrointestinal diseases because of its diverse range of bioactive compounds, including β-glucans, diterpenoids, and phenolic compounds. Erinacines are cyathane diterpenoids found on the mycelium of HE, and hericenones are benzyl alcohol derivatives that can be extracted from the fruiting body. Erinacines can cross the blood–brain barrier via passive diffusion and exert neurotropic, neuroregenerative, and neuroprotective effects by affecting neurotrophins. Neurotrophins, such as nerve growth factor (NGF), BDNF, and GDNF, support neurite outgrowth and development and protect neurons from damage. However, they are large molecules that cannot cross the blood–brain barrier. Thus, there is particular interest in developing pharmaceutical agents that can stimulate the production of neurotrophins for the management of neurodegenerative diseases [44,45].

A limitation to the use of nutraceuticals containing HE worldwide has been the fact that dehydrated mycelium powder is considered a novel food in the European Union (EU). Therefore, pre-market authorization in accordance with Regulation EU 2015/2283 is required before it can be placed as food on the EU market. Currently, only fruiting body extracts rich in hericenones are permitted, despite the fact that erinacines, known for their ability to stimulate neurotrophins, can be found in greater concentrations in the mycelia [195].

### 7.2. Preclinical Studies

*In vitro* studies have shown that erinacines present on HE can stimulate the synthesis of NGF; however, this could not be induced in neurons alone and was only possible when they were administered to glial cells. Erinacines A, C, and S enhance neurite outgrowth in NGF-responsive models, but whether they are able to potentiate NGF signaling or they mimic neurotrophin activity is still under investigation [44,196]. The ability of hericenones to also stimulate NGF biosynthesis is still controversial [197].

In a systematic review of preclinical models, Spangenberg et al. included studies that evaluated the neuroprotective effects of different erinacines, with most of the available evidence focusing on erinacine A, S, and C. Some studies investigated the effects of HE mycelia (HEM), whereas others investigated the effects of mycelial extracts (HEME); overall, the extraction methods, concentrations, and cell lines used varied significantly. Each erinacine appears to have a different role. The neuroprotective effects of erinacine A and HEME treatment were mostly associated with an inhibitory effect on NF-κΒ activation. In rodent PD models, erinacine A administration improved motor function by reducing dopaminergic neurotoxicity and protecting against microglia-mediated neuroinflammation by decreasing pro-inflammatory cytokine expression and ROS production in the midbrain. Tsai-Teng et al. showed that HEME which was higher in erinacine A concentration was more potent at reducing Aβ plaque burden than HEM in an AD model [198]. In another study, erinacine A improved spatial learning and memory by reducing Aβ plaque formation but had moderate benefits in the neurologic deficits in a brain ischemia mouse model [44].

Erinacine C administration has been associated with an increase in antioxidant enzyme levels, whereas treatment with erinacine S induced the accumulation of neurosteroids, in particular, pregnenolone and progesterone, which promote neurogenesis, neurite outgrowth, and protect neurons against apoptosis. However, very high concentrations of erinacines A, C, and S decreased cell viability in human neuroblastoma SH-SY5Y cells [44].

### 7.3. Clinical Studies

A recent systematic review included 10 clinical studies (5 RCTs, 3 pilot studies, 1 cohort study, and 1 case report) that explored the benefits and side effects of HE in conditions such as cognitive dysfunction and mood disorders. The type and dose of formulation, duration of intervention, and outcome measures in these studies varied [199].

We identified three studies that focused on the acute effects of HE administration [200,201]. Doherty and associates conducted a pilot study in young, healthy adults to determine the acute and chronic effects of HE supplementation on cognition and mood. Forty-one volunteers aged 18–45 years were assigned to receive 1.8 g of lion’s mane mushroom (SO-DSX1^®^, Sempera Organics Inc., Morgan Hill, CA, USA) capsules or a placebo. Cognitive function tests included immediate and delayed word recall, numeric working memory, choice reaction time, the Stroop task, peg and ball, and delayed word recognition. These tests were designed to measure effects on attention, executive function, working memory, and episodic memory and were delivered via a computerized mental performance assessment system (COMPASS). Mood and psychological state were assessed using the Stress Visual Analog Scale (VAS), the Visual Analog Mood Scale and the Perceived Stress Scale. Participants performed significantly faster on the Stroop task 1 h after administration of lion’s mane supplementation but performed less accurately on the immediate word recall test, in contrast to the placebo group, which had fewer errors in immediate word recall. After 28 days of supplementation, some improvement was noted regarding subjective stress scores in the intervention arm; however, in the placebo group, delayed word recall after 28 days was better than that on baseline [201].

La Monica et al. conducted a randomized, double-blind, placebo-controlled, nested within-subject crossover study that examined the acute effects of a single dose of 650 mg guayusa extract vs. 1 g Nordic-grown Lion’s Mane vs. placebo in healthy adults. In this study, the authors utilized the Go/No-go test to assess inhibitory responses, the Serial Sevens test, which evaluates attention concentration and working memory, and the N-back test for working memory. Subjective questionnaires were used to evaluate mood, focus, stress, and happiness. Contrary to the previous study by Docherty et al., in this study, the assessment was conducted both in the first and second hour after ingestion. Lion’s mane improved reaction time to the stimulatory response at 120 min during the Go/No-go test, improved reaction time at the N-back test from 60 to 120 min, and improved the number of attempted responses during the serial sevens test after 2 h, potentially showing a delayed response after ingestion. Subjective ratings of happiness and “getting the most out of everything” also improved. This study showed that a single dose of lion’s mane can improve reaction time, working memory, attention, and self-perceived happiness compared to baseline, which could indicate that long-term intake is not necessary for the positive effects, although remodeling of nerve structures likely requires a longer duration or higher doses [200]. A very recent double-blind, randomized, placebo-controlled, cross-over study by Surendran et al. investigated the acute effects of HE on cognition and mood in 18 healthy young adults aged 18 to 25 years in the UK. The intervention involved a 250 mL drink comprising 3 g of HE fruiting body extract. The results failed to detect any effect on cognition as determined by the global composite measure; however, acute consumption of the intervention drink resulted in improved psychomotor skill and manual dexterity as evaluated on the grooved pegboard test at 90 min. However, it appeared to impair performance on the Flanker and Trail making B tests, which assess executive function [202].

Despite the fact that neuroprotective erinacines are primarily found in the mycelia of HE, in a pilot study, fruiting body powder of HE, which contains primarily hericenones, also had a beneficial effect on cognitive function. This was a double-blind, parallel-group, placebo-controlled trial in which 30 Japanese patients, aged 50–80 years, diagnosed with mild cognitive impairment were randomized to tablets containing 96% fruiting body dry powder of HE (3 g total daily dose) for 16 weeks or a placebo. The intervention group showed improved scores of MCI during the intervention period compared to the placebo group; however, the results decreased significantly after discontinuation at the 4-week follow-up, hinting that the beneficial effect of HE on mild cognitive impairment is reversible after discontinuation [203]. In another randomized double-blind, placebo-controlled, parallel-arm comparative study on 31 healthy adults aged >50 years old, supplements of HE fruiting body powder improved MMSE scores [204]. A double-blind, placebo-controlled RCT evaluated the effects of daily erinacine A-enriched HE supplementation (EAHE; 3.44 mg/day) over 8 weeks in healthy adults over 55. Adjusted analyses revealed significant improvements in cognitive performance in the intervention group, accompanied by an increase in serum BDNF, whereas biochemical markers and neuropeptide Y remained unchanged. Modest changes in gut microbiota diversity were observed, although overall composition was largely stable, likely reflecting the short intervention period and limited sample size [205].

Chen Li et al. conducted a one-year, double-blind, randomized, placebo-controlled pilot trial to assess whether EAHE supplementation could slow progression in patients with mild AD. Participants received EAHE (three 350 mg capsules/day, 5 mg/g erinacine A) or placebo for 49 weeks and were monitored with cognitive assessments, biomarkers, ophthalmic exams, and neuroimaging. Compared to placebo, the EAHE arm showed improved MMSE scores, better Instrumental Activities of Daily Living (IADL) outcomes, reduced Cognitive Abilities Screening Instrument (CASI) decline, less deterioration in blood biomarkers, and less structural disorganization on neuroimaging. Although this was a small pilot study, its study design allowed the evaluation of clinical, biochemical, and imaging data pertinent to AD [206].

Mood improvements were observed in another study using powdered fruiting bodies of HE cookies. Using a randomized, double-blind, placebo-controlled design, scientists investigated the effects of HE on perimenopausal symptoms, depression, sleep quality, and indefinite complaints. A total of 30 women were randomized to receive four HE cookies (0.5 g powdered fruiting body of HE) or placebo cookies each day, for a period of 4 weeks. The authors noted improvements in depressive symptoms and anxiety scores post-intervention compared to the baseline; however, no significant difference was observed between intervention and placebo arms [207]. Vigna and associates assessed the effects of HE supplementation (80% bulk mycelia, 20% fruiting body extract) on mood disorders, BDNF, and pro-BDNF serum concentrations in volunteers with overweight or obesity on a low-calorie diet regimen. After 8 weeks of HE supplementation, levels of pro-BDNF were increased, BDNF concentrations remained unchanged and an improvement was noted in depressive symptoms, anxiety and disordered sleep [208].

## 8. Flavonoids

There are hundreds of polyphenolic compounds in nature, including flavonoids, found mostly in green plants and seeds, with multiple beneficial actions [209,210]. The best well-known flavonoids include apigenin, diosmin, luteolin, naringin, pycnogenol, quercetin, and rutin. The number of phenolic groups dictates their well-known antioxidant effects, but also constitutes a problem for those with phenol intolerance due to metabolic enzyme polymorphisms.

In the context of neurodegenerative diseases, flavonoids have been reported to exert protective effects through mechanisms such as attenuating neuroinflammation, reducing oxidative stress, and modulating neuronal signaling pathways [211,212]. Greater dietary intake of polyphenols has been associated with a reduced risk of cognitive decline and neurodegeneration, potentially through improvements in vascular function, synaptic plasticity, and mitochondrial health [213,214]. Additionally, flavonoids have been implicated in memory preservation, with evidence suggesting protective roles against amnesia via cholinergic modulation and anti-inflammatory actions in the brain [212]. Anthocyanins have also shown promise in improving cognitive performance in both healthy and cognitively impaired individuals, further supporting their role across the lifespan in maintaining brain health [215].

### 8.1. Quercetin

Quercetin is a naturally occurring flavonoid abundant in fruits and vegetables, such as apples and onions [216]. It exerts antioxidant and anti-inflammatory effects by modulating several redox- and inflammation-related pathways, including activation of the NRF2/ARE (antioxidant response element) and inhibition of NF-κB signaling and the neutrophil-to-lymphocyte ratio (NLR) family, NLRP3 [26,27,28,29]. The antioxidant effects of quercetin reflect the modulation of several oxidative stress biomarkers including glutathione (GSH), glutathione reductase (GR), glutathione-S-transferase (GST), glutathione peroxidase (GPx), superoxide dismutase (SOD), catalase (CAT), and malondialdehyde (MDA) [217]. Preclinical studies have demonstrated that quercetin attenuates stress-induced behavioral and biochemical alterations, partly through modulation of corticotropin-related stress signaling in the brain [218]. In addition, quercetin exhibits neuroprotective effects by suppressing oxidative and nitrosative stress-mediated neuroinflammation and apoptosis pathways implicated in depression [219,220,221], effects that may occur independently of the hypothalamic–pituitary–adrenal (HPA) axis [222].

Beyond mood-related outcomes, experimental models indicate that quercetin may also improve cognitive function and protect against neurodegenerative processes. In animal models of AD, quercetin reduces Aβ accumulation, attenuates neuro-inflammation, while improving learning and memory performance [223]. Additional studies suggest that quercetin modulates microglial activation and enhances endogenous antioxidant defenses, further contributing to its neuroprotective properties [224].

Despite strong preclinical evidence, clinical research remains limited. Two randomized, double-blind, placebo-controlled trials investigated the effects of quercetin on cognitive function in older adults. In the first, 70 healthy individuals aged 60–79 years old were randomized to receive quercetin-rich onion providing approximately 50 mg/day of quercetin or a quercetin-free placebo for a period of 24 weeks [225]. The quercetin arm demonstrated improvements in the MMSE scores and emotional function, suggesting reduced age-related cognitive decline and improved mood-related outcomes [225]. In the second trial, 80 older adults (60–75 years old) supplemented with a beverage containing 110 mg/day of quercetin glycosides for a period of 40 weeks [226]. The intervention resulted in improved reaction time, suggesting a potential attenuation of age-related decline in cerebral blood flow and brain activity, possibly through stress reduction and inhibition of Aβ accumulation [226]. Further well-designed clinical trials are required to clarify the potential role of quercetin in supporting brain health and cognitive function.

### 8.2. Apigenin

Apigenin (4′,5,7-trihydroxyflavone) is a naturally occurring flavonoid commonly found in plants of the *Apiaceae* family (e.g., celery, carrot, and parsley) and in chamomile tea derived from the dried flowers of *Matricaria chamomilla* [227]. In most dietary sources apigenin predominantly exists as a glycoside, such as apiin in parsley and celery or apigenin 7–O–glucoside in chamomile, which are enzymatically hydrolyzed in the gut to release the bioactive aglycone. Other foods containing measurable apigenin glycosides include onions, citrus fruits (oranges, grapefruit), tea, and wheat sprouts [227,228].

Apigenin modulates GABAergic and glutamatergic neurotransmission in vitro through effects on GABA_A_ and NMDARs, and has been reported in pharmacological studies to influence central nervous system activity [229]. Research has shown that it can improve cognitive function in AD models by reducing Aβ burden, inhibiting the amyloidogenic pathway (downregulating BACE1; beta-site APP cleaving enzyme 1 and β-cleavage C-terminal fragment (β-CTF), scavenging ROS, enhancing antioxidant enzymes (SOD, GPx), and restoring the ERK/CREB/BDNF (extracellular signal-regulated kinase; cAMP response element-binding protein) neurotrophic pathway [230]. In neuronal cell models, apigenin protects against Aβ- and copper-induced toxicity by maintaining redox balance, preserving mitochondrial function, inhibiting MAPK signaling, and reducing apoptosis [231]. Apigenin also preserves neurovascular integrity, improves cerebral blood flow, and supports cholinergic function (increasing ACh and reducing AChE activity) [232]. In models of depression, apigenin reverses behavioral deficits induced by chronic corticosterone or forced swimming, likely through upregulation of hippocampal BDNF, modulation of dopaminergic, serotonergic, and noradrenergic systems, and normalization of HPA axis activity [233,234,235,236], reduces elevated serum corticosterone, and restores platelet adenylyl cyclase activity [236]. Apigenin’s antidepressant-like effects are also supported by evidence that it attenuates pro-inflammatory cytokine production (IL-1β, TNF-α) and suppresses inducible NOS and cyclooxygenase-2 (COX-2) expression via inhibition of NF-κB signaling in models of inflammation-induced depressive behavior [235].

Very few clinical studies evaluating apigenin exist in the literature, mostly from *Matricaria chamomilla* L., involving people with anxiety disorder [237,238]. In a double-blind RCT of 57 outpatients with mild-to-moderate generalized anxiety disorder, oral chamomile extract (220 mg capsules standardized to 1.2% apigenin) administered over 8 weeks significantly reduced Hamilton Anxiety Rating-A scores compared with placebo, demonstrating efficacy and tolerability as a potential anxiolytic [237]. A crossover RCT in 100 patients with migraine without aura evaluated a topical chamomile oleogel standardized for apigenin content (0.233 mg/g). Application to the temporal and forehead areas during attacks significantly reduced pain sensation, nausea, vomiting, photophobia, and phonophobia compared to placebo. Notably, 2 h after application, 29.2% of attacks were pain-free with chamomile versus 2.1% with placebo, and sustained relief over 24 h was observed in 74.3% of attacks versus 10.4% for placebo [238].

### 8.3. Diosmin

Diosmin (diosmetin 7-O-rutinoside) is a flavone glycoside derived from citrus fruits, commonly used for hemorrhoids and leg ulcers caused by poor blood circulation [239,240]. It protects neurons from CPF-induced oxidative injury by decreasing MDA content and increasing GSH, GST, and SOD levels through PPAR-γ upregulation [241]. Diosmin also reduces chlorpyrifos-induced neuroinflammation by lowering myeloperoxidase activity and pro-inflammatory cytokines (tumor necrosis factor α; TNF-α, IL-1β, and IL-6) via downregulation of NF-κB/AP-1 (activator Protein 1) signaling (c-FOS and c-JUN) [241]. It shows promising neuroprotective effects in preclinical models of AD. In 3xTg-AD mice, oral diosmin decreased cerebral soluble Aβ and Aβ oligomer levels, inhibited tau hyperphosphorylation, and improved cognitive deficits by inhibiting GSK-3α/β and upregulating transient receptor potential canonical 6, which selectively suppresses APP γ-secretase activity without affecting Notch processing [242]. Its main metabolite, diosmetin, similarly reduced Aβ generation, tau phosphorylation, and pro-inflammatory microglial activation in vitro. In Aβ25-35-induced AD rat models, diosmin enhanced learning and memory, decreased hippocampal neuronal apoptosis, and lowered pro-inflammatory cytokines (IL-1β, IL-6, TNF-α), effects associated with activation of the phosphatidylinositol-3-kinase (PI3K)/protein kinase beta (Akt) pathway [243]. It has also demonstrated neuroprotective effects in preclinical models of traumatic brain injury, improving cognitive deficits and hippocampal long-term potentiation while reducing pro-inflammatory TNF-α levels [244,245]. Up to date, no clinical studies have evaluated the role of diosmin administration for brain health.

### 8.4. Luteolin

**Figure 4:**
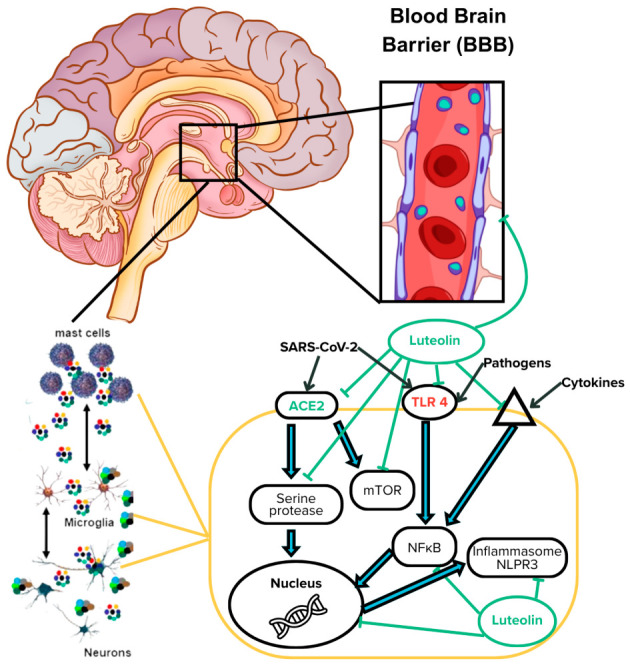
Diagrammatic representation of the beneficial actions and target points of luteolin: stabilization of the blood–brain barrier (BBB), inhibition of mast cells, microglia and neuroprotection, via inhibition of activation of different surface receptors, as well as inhibition of numerous downstream stimulus–response coupling events. ACE2: Angiotensin-converting enzyme 2; BBB: Blood–brain barrier NF-κB: Nuclear Factor Kappa B; NLRP3: NOD-, LRR- and pyrin domain-containing protein 3; TLR-4: Toll-like receptor 4. Orange oval represents a generic mast cell, microglial cell or neuron as indicated by the corrsponding lines.

Luteolin (3′,4′,5,7-tetra-hydroxy-flavone) is a flavone naturally occurring in several plant-based foods, including celery, carrots, dried parsley, broccoli, green chili, spinach, cabbage, thyme, and fresh peppermint [246]. Preclinical studies suggest that luteolin exerts neuroprotective effects through a combination of anti-inflammatory and antioxidant mechanisms [247] (Figure 4).

In animal models, cerebral hypoperfusion activates NF-κB, increases the expression of BACE1, and elevates Aβ concentrations in the cortex and hippocampus; luteolin administration attenuated these pathological changes [248,249]. In 3×Tg-AD mice treated with luteolin for three weeks, spatial learning improved, and memory deficits were ameliorated. These effects were accompanied by inhibition of glial fibrillary acidic protein (GFAP), reduction in neuroinflammatory markers (TNF-α, IL-1β, IL-6, nitric oxide, COX-2, and iNOS), and decreased expression of endoplasmic reticulum stress markers glucose-related protein 78 (GRP78) and inositol-requiring transmembrane kinase/endoribonuclease 1α (IRE1α) in brain tissues in a dose-dependent manner [250]. It has also been found to improve insulin sensitivity by enhancing hippocampal insulin signaling in Aβ25-35-induced AD rat models [251]. Similarly, in a rodent model of Parkinson’s disease, luteolin reduced dopaminergic neuronal loss and improved motor function by downregulating the TLR4 signaling pathway, inhibiting NF-κB activation, and shifting microglia toward an anti-inflammatory (M2) phenotype [252] (Figure 4). Additionally, luteolin exerts antioxidant properties, scavenging ROS, while preventing decreases in mitochondrial activity as well as in the antioxidant enzymes CAT and GSH in ROS-insulted primary neurons [253]. The antioxidant activity of luteolin is nested in its glycosidic group, which helps in removing reactive nitrogen species (RNS) and ROS [254].

Luteolin can enter the brain and reduce microglial activation [255], particularly when stimulated by LPS [256]. Luteolin further protected against propionate-induced organ damage and ASD-like behavior in animal models [257,258]. Luteolin also inhibits activation of the NLRP3 inflammasome (Figure 4) [30,31]. The structural luteolin analog, tetra-methoxy-flavone, is even more potent than luteolin in inhibiting activated microglia [259].

#### Clinical Studies

Emerging evidence suggests that luteolin-containing formulations may exert beneficial effects in inflammation-associated conditions [211].

Use of the luteolin-containing dietary supplement (NeuroProtek^®)^ in two open-label studies of children with ASD resulted in significant clinical improvement [260,261] (Table 5). Moreover, use of this dietary supplement reduced pro-inflammatory serum cytokines IL-6 and TNF, particularly in a high-inflammatory subgroup, which correlated with the greatest gains in adaptive behavior, including 6–10 months developmental improvements in communication, social skills, and daily living activities [262]. Unfortunately, many cheap preparations of luteolin are of low purity, fail to disclose the source, while in order to reach the ideal daily dose multiple capsules or tablets are required [263]. It should be noted that high amounts (>2000 mg/day) of any flavonoid in powder form (<10% absorption) can disrupt liver and microflora catabolic enzymes [264,265].

Some clinical studies have also investigated luteolin in combination with PEA in patients with neurological conditions such as frontotemporal dementia, acute ischemic stroke, and in individuals with long COVID experiencing chronic olfactory dysfunction (“brain fog”) [150,154,266]. Benefits have been reported in cognitive function and neuropsychiatric symptoms, including improvements in cognitive function, daily living activities, olfactory identification, and mental clarity, although some studies suggest that the additional clinical benefit over olfactory training alone may be modest [267]. In addition, a combination of luteolin and quercetin has been studied in children with ASD [261], where treatment was associated with improvements in behavioral symptoms and communication in some patients. Finally, a case report demonstrated the efficacy of luteolin in post-Lyme syndrome-associated polyneuropathy, where NeuroProtek^®^ in combination with intravenous immunoglobulin (IVIG) led to progressive improvements in neuropathy, fatigue, cognitive symptoms, and “brain fog,” culminating in complete symptom resolution after nine months of therapy [268].

Moreover, recent human research has explored the effects of luteolin in combination with other polyphenols on physical performance. Supplementation with mangiferin and luteolin for both short-term (48 h) and longer-term (15 days) periods was shown to enhance exercise performance in men, suggesting potential synergistic effects on energy metabolism, oxidative stress reduction, and muscle function [269].

Overall, clinical evidence suggests that luteolin, alone or in combination with PEA or quercetin, exerts anti-inflammatory, neuroprotective, and immunomodulatory effects across a spectrum of pediatric and adult neuropsychiatric and neuroinflammatory conditions. While findings in ASD, dementia, and post-infectious olfactory disorders are encouraging, further well-designed, placebo-controlled clinical trials are required to fully define its therapeutic potential, optimal dosing, and long-term safety.

**Table 5:** Clinical applications of luteolin and co-ultramicronized PEA–Luteolin formulations in neuroinflammatory and post-infectious conditions.

Compound	Model/Population	N	Dosage	Mechanism of Action	Key Outcomes	Reference
Co-ultramicronized PEA + Luteolin	Patients with frontotemporal dementia	48	PEA 700 mg/day + luteolin 70 mg/day	Anti-inflammatory; modulation of neuroinflammation via PPAR-α and CB1 receptors; reduction in microglial activation and synaptic dysfunction	↓ decline in global disease severity & functional deterioration	[150]
Co-ultramicronized PEA + Luteolin	Patients with acute ischemic stroke	60	PEA 700 mg/day + luteolin 70 mg/day	Neuroprotective; anti-inflammatory; reduction in secondary neuronal damage and oxidative stress	↑ independence in daily living, ↑ cognitive recovery	[154]
Luteolin (NeuroProtek^®^ lioposomal formulation in olive pomace oil)	Children with ASD (open-label pilot)	37	1 softgel/10 kg body weight, each containing luteolin 100 mg, quercetin 70 mg, and rutin 30 mg	Mast cell stabilization; ↓ neuroinflammation; inhibition of cytokine release (IL-6, TNF-α)	Improved behavior, communication, and attention; reduction in inflammatory symptoms	[260]
Luteolin (NeuroProtek^®^ liposomal formulation in olive pomcae oil	Children with ASD (open-label pilot)	50	1 softgel/10 kg body weight, each containing luteolin 100 mg, quercetin 70 mg, and rutin 30 mg	Anti-inflammatory; inhibition of mast cells and microglia; modulation of neuroimmune signaling	Behavioral improvements (social interaction, irritability); good tolerability	[261]
Luteolin (NeuroProtek^®^ liposomal formulation in olive pomcae oil	Children with ASD (open-label pilot)	40	1 softgel/10 kg body weight, each containing luteolin 100 mg, quercetin 70 mg, and rutin 30 mg	Anti-inflammatory; mast cell stabilization; inhibition of microglial activation; ↓ pro-inflammatory cytokines (IL-6, TNF)	Significant reduction in IL-6 and TNF; identification of high-inflammatory subgroup; greatest behavioral improvement in this subgroup (↑ communication, social interaction, daily living skills; ~6–10 months developmental gains)	[262]
Co-ultramicronized PEA + Luteolin (±olfactory training)	Patients with long COVID (olfactory dysfunction, “brain fog”)	69	PEA 700 mg/day + luteolin 70 mg/day	Anti-inflammatory; modulation of neuroinflammation via PPAR-α activation; reduction in microglial and mast cell activation; neuroprotective effects on olfactory pathways	Improvement in olfactory function (odor identification ↑ by 10.7 ± 2.6 at 3 months, *p* < 0.0001); reduction in parosmia (*p* < 0.0001); improvement in mental clouding/brain fog (*p* = 0.02); enhanced memory function	[266]
Co-ultramicronized PEA + Luteolin (±olfactory training)	Patients with post-viral olfactory dysfunction (mostly COVID-19)	50	PEA 700 mg/day + luteolin 70 mg/day	Anti-inflammatory; modulation of neuroinflammation via PPAR-α; reduction in mast cell activation; shift in microglia toward anti-inflammatory (M2) phenotype; inhibition of NF-κB, STAT3, and AP-1 signaling	Within-group improvement in olfactory function (TDI score ↑, *p* = 0.031; discrimination ↑, *p* = 0.049) in PEA–Luteolin arm; however, no difference between arms was recorded in overall clinical improvement; limited added benefit beyond olfactory training	[267]
Luteolin (NeuroProtek^®^ formulation) + IVIG)	Post-Lyme syndrome-associated polyneuropathy	1 (case report)	1 softgel/10 kg body weight, each containing luteolin 100 mg, quercetin 70 mg, and rutin 30 mg	Anti-inflammatory flavone; mast cell and microglia inhibition; immunomodulation in combination with IVIG; neuroprotection; reduction in neuroinflammation and microglial activation	Progressive improvement in neuropathy, fatigue, “brain fog,” and cognitive symptoms; complete symptom resolution after 9 months of combination therapy; no adverse effects reported	[268]
PEA + luteolin	Patients with frontotemporal dementia	17	PEA 700 mg/day + luteolin 70 mg/day	Neuroinflammation modulation; ↑ GABAergic transmission; ↓ microglial activation	Improved frontal lobe function, electrophysiological changes (EEG HF oscillations)	[270]
Mangiferin + Luteolin	Healthy men (exercise performance clinical trial)	48	Luteolin ~100 mg/day	Antioxidant; free radical scavenging; ↓ superoxide-producing enzymes; activation of antioxidant gene pathways	Improved sprint exercise performance; enhanced metabolic and oxidative efficiency	[269]

ASD: autism spectrum disorder; EEG: electro-encephalography; HF: high frequency; IL: interleukin; IVIG: intravenous immunoglobulin; M2: anti-inflammatory microglia phenotype; NF-κB: nuclear factor kappa-light-chain-enhancer of activated B cells; PEA: palmitoylethanolamide; PPAR-α: peroxisome proliferator-activated receptor alpha; STAT3: signal transducer and activator of transcription 3; TDI: threshold, discrimination, identification (olfactory test score); TNF: tumor necrosis factor; ↑: increased; ↓: decreased.

### 8.5. Naringin

Naringin (2,3-dihydro-5,7-dihydroxy-2-(4-hydroxyphenyl)-4H-1-benzopyran-4-one) is a bioflavonoid commonly found in citrus fruits such as grapefruits, lemons, and oranges [271]. A systematic review and meta-analysis of rodent studies revealed that naringin supplementation improved cognitive performance and reduced oxidative stress by increasing antioxidant enzyme activity, improving mitochondrial function, and modulating neuronal enzymes involved in neurotransmission [272].

Experimental studies suggest that naringin exerts neuroprotective effects through multiple pathways, including enhancement of insulin signaling in the brain and modulation of πeroxisome proliferator-activated receptor gamma (PPAR-γ) pathways [273]. In animal models, chronic administration of naringin improved cognitive performance and reduced mitochondrial oxidative damage, AChE activity, and brain aluminum accumulation in aluminum-treated rats [274].

The aglycone form, naringenin, also demonstrates neuroprotective properties according to research. In APP/PS1 transgenic mice, naringenin treatment significantly reduced Aβ deposition in the hippocampus and cortex and attenuated neuroinflammation through suppression of the MAPK signaling pathway, which is linked to GSK-3β activation and tau phosphorylation [275]. In addition, synthetic naringenin-O-carbamate derivatives (e.g., compound 3c) have shown potent inhibition of Aβ aggregation, the ability to disaggregate Cu^2+^-induced Aβ_1–42_ aggregates, and significant neuroprotective activity, highlighting the relevance of targeting metal-associated amyloid aggregation in AD [276]. Further experimental evidence from an aluminum chloride (AlCl_3_)-induced AD-like rat model demonstrated that naringin ameliorated cerebellar dysfunction, oxidative stress, and behavioral impairments. AlCl_3_ exposure caused deficits in spatial learning and motor coordination, increased lipid peroxidation, and depletion of endogenous antioxidants such as GSH, whereas naringin treatment significantly mitigated these alterations [277]. Despite these promising preclinical findings, clinical studies evaluating the effects of naringin on brain health are currently lacking.

### 8.6. Pycnogenol

Pycnogenol is a standardized extract obtained from the bark of the French maritime pine (*Pinus pinaster*) and consists primarily of flavonoid compounds, including monomers such as catechin, epicatechin, and taxifolin, as well as oligomeric procyanidins/proanthocyanidins. It is widely recognized for its potent antioxidant properties [278].

Preclinical evidence consistently demonstrates that Pycnogenol exerts antioxidant, anti-inflammatory, vasoprotective, and neuroprotective effects across a range of in vitro and in vivo models, although these effects appear to be dose- and context-dependent. In models of cerebral ischemia, pycnogenol preserved neuronal viability, reduced oxidative stress markers (ROS, MDA), restored antioxidant enzyme activity (SOD, CAT), and attenuated neuroinflammatory signaling via inhibition of NF-κB and extracellular signal-regulated kinases 1 and 2 (ERK1/2) pathways, ultimately improving memory and limiting hippocampal neuronal loss [279,280,281]. In AD models, it reduced Aβ plaque burden and improved spatial memory when administered prior to pathology onset, suggesting a potential role in early intervention rather than disease reversal [282]. Similarly, in PD models, Pycnogenol protected dopaminergic neurons and improved motor outcomes through suppression of oxidative stress and pro-inflammatory mediators, including TNF-α, IL-1β, iNOS, and cyclo-oxygenase-2 (COX-2) [283]. Mechanistic studies further demonstrate that Pycnogenol modulates microglial activation, inhibiting NF-κB and AP-1 signaling while reducing cytokine production [284,285]. Beyond the central nervous system, Pycnogenol enhances endothelial antioxidant defenses and promotes nitric oxide-mediated vasodilation, supporting its role in cerebrovascular health [286,287], while also improving metabolic and oxidative stress parameters in diabetic models [288]. Notably, some *in vitro* studies suggest potential toxicity at supraphysiological concentrations, whereas *in vivo* studies indicate protective effects, highlighting the importance of dose optimization and physiological relevance [289]. Collectively, these findings support a multimodal mechanism of action, but also underscore the need for careful translation into clinically relevant dosing strategies.

Clinical studies have explored its potential neuroprotective and cognitive effects. In patients with TBI, supplementation with French maritime pine bark extract (150 mg/day) for 10 days significantly reduced inflammatory markers, including IL-6, IL-1β, and C-reactive protein (CRP), and improved clinical severity scores [290]. Several RCTs have investigated the effects of pycnogenol in children with attention-deficit hyperactivity disorder (ADHD). Supplementation with approximately 1 mg/kg of body weight each day for a period of one month improved hyperactivity, inattention, and visual–motor coordination [291]. These effects were accompanied by significant improvements in oxidative stress biomarkers, including decreased glutathione disulfide (GSSG), increased GSH concentrations, and an improved GSH/GSSG ratio [292], as well as reductions in 8-oxoguanine, a biomarker of oxidative DNA damage [293,294]. In another RCT in pediatric patients with ADHD, pycnogenol administered at 20 mg/day for individuals weighing <30 kg and 40 mg/day for those ≥30 kg (with 20 mg/day during the first two weeks) improved total and hyperactivity/impulsivity scores and increased CAT activity [291].

In healthy adults, supplementation with pycnogenol (150 mg/day) for three months improved aspects of memory performance, particularly the quality of working memory as assessed by spatial and numeric working memory accuracy scores [295].

Several additional randomized controlled trials have been designed to further investigate the health effects of pycnogenol. One ongoing RCT is evaluating supplementation with pycnogenol (150 mg/day) in relation to aging-related outcomes, including fecal biomarkers associated with gut microbiota composition [296]. Finally, another ongoing RCT is investigating pycnogenol effects in patients with ADHD, administering 20 mg/day to participants weighing <30 kg and 40 mg/day to those ≥30 kg, corresponding to approximately 1 mg/kg/day, with the first two weeks standardized at 20 mg/day [297].

### 8.7. New Flavonol Molecules

Recent work has started to move beyond the well-known flavonoids and has drawn attention to a newer set of molecules, such as prenylated flavonoids, biflavonoids, and microbiota-derived metabolites, that seem to have stronger and more nuanced biological effects [298,299,300]. What makes these compounds particularly interesting is not just their antioxidant capacity, but their ability to actively regulate cell signaling pathways that are tightly linked to oxidative stress. Rather than acting simply as direct scavengers of ROS, many of these emerging flavonoids appear to work by modulating key systems, such as the Kelch-like ECH-associated protein 1 (KEAP1)–NRF2 pathway, NF-κB, and PI3K/Akt, effectively “reprogramming” how cells respond to stress [298,299,300].

This becomes especially relevant in the context of brain health, where oxidative imbalance and dysregulated signaling are central features of neurodegenerative conditions [21]. Some of these newer flavonoid forms, particularly prenylated structures and low-molecular-weight metabolites produced by the gut microbiota (including daidzein, enterolactone, protocatechuic acid, urolithins, and γ-valerolactones among others) also show improved bioavailability and may cross the blood–brain barrier more efficiently, thereby addressing a major limitation of classical flavonoids [301,302,303,304,305]. For example, chemically modified or semi-synthetic molecules such as 8-chloro-3′,4′,5,7-tetrahydroxyflavone have been shown to more effectively control the oxidative burst in activated neutrophils while also modulating downstream inflammatory pathways [306]. Similarly, acetylated isoflavone glycosides, such as 6″-O-acetylgenistin and 6″-O-acetyldaidzin strongly suppress lipid peroxidation, demonstrating that subtle chemical modifications can tune both redox properties and interactions with molecular targets involved in oxidative stress [306]. Certain less-common natural flavanols, such as (−)-(2R,3R)-5,7-dimethoxy-3′,4′-methylenedioxy-flavan-3-ol from cinnamon, activate Nrf2 and enhance endogenous antioxidant defenses, illustrating how ROS control can be coupled to well-defined signaling pathways [307]. Flavonoid mixtures, such as the n-butanol fraction from *Polygonum hydropiper*, protect immune cells from viral infection by reducing ROS, increasing antioxidant enzymes, and simultaneously activating the PI3K/Akt–NRF2/HO-1 (heme oxygenase-1) axis, while balancing histone deacetylase/histone acetyltransferase (HDAC/HAT) activity, providing a clear example of dual functional roles [308]. Other natural flavonoids, including oroxylin A, activate antioxidant NRF2/ARE programs while inhibiting pro-NF-κB signaling, and prenylated flavonoids like xanthohumol modulate oxidative stress in conjunction with cardiovascular signaling through the phosphatase and TENsin homolog (PTEN)–Akt/mTOR pathway [308,309]. In addition, components like isorhamnetin have been shown to enhance treatment efficacy, often acting synergistically with other therapeutic agents [301,302]. Rather than exerting isolated effects, isorhamnetin appears to modulate overlapping pathways involved in oxidative stress and cell signaling, thereby amplifying overall cellular responses [301,302].

Thus, flavonoids should no longer be viewed simply as dietary antioxidants, but rather as multi-target modulators with significant potential in maintaining brain function and possibly slowing the progression of neurological disorders.

### 8.8. Bioavailability

Despite their promising biological activities, flavonoids have limitations, including poor and highly variable bioavailability, which significantly constrains their clinical translation [310]. Most dietary flavonoids are present as glycosides and undergo extensive phase II metabolism (glucuronidation, sulfation, and methylation) in the intestine and liver, resulting in low circulating concentrations of the parent compounds [310,311]. Moreover, their limited solubility, rapid elimination, and restricted permeability across the blood–brain barrier further reduce their effective concentrations at target sites within the central nervous system [303]. Importantly, only a small fraction of ingested flavonoids is absorbed in the small intestine, while the majority reaches the colon, where it is metabolized by the gut microbiota into low-molecular-weight phenolic metabolites, which may represent the biologically active forms [312]. These metabolites often exhibit improved bioavailability and blood–brain barrier penetration compared with their parent compounds [312]. To address these limitations, recent research has focused on novel delivery systems and structural modifications to enhance absorption and stability. Overall, improving bioavailability remains a critical step for translating the promising mechanistic properties of flavonoids into consistent clinical outcomes.

## 9. Olive Oil Polyphenols

Olive oil polyphenols have potent antioxidant, anti-inflammatory and neuroprotective properties [313]. Within brain regions such as the hippocampus and cortex, olive polyphenols exert several central effects, including modulation of neuroimmune tone through regulation of microglial activity, activation of the KEAP1/Nrf2/ARE antioxidant pathway, and support of neurotrophic signaling pathways involved in the memory–cognition–mood axis, including BDNF, tropomyosin receptor kinase B (TrkB), and cAMP response element-binding protein (CREB) [314]. Furthermore, there is evidence to suggest interactions of olive oil polyphenols and dopaminergic metabolism [314].

### 9.1. Oleuropein

Oleuropein is a major phenolic compound of olive products and a precursor of HT. Both compounds cross the BBB [315], however, oleuropein—in its free form—exhibits relatively low bioavailability due to its hydrophobic structure, higher molecular weight, and partial degradation in the colon [315,316]. Absorption is thought to occur partly through glucose transporters [315].

Several experimental studies have demonstrated the neuroprotective potential of oleuropein. In a TBI model, oleuropein (50–100 mg/kg, i.p.) reduced the expression of matrix metalloproteinase-9, glial fibrillary acidic protein (GFAP), and high mobility group box-1, particularly during the early phase and 24 h post-TBI induction [317]. In a D-galactose-induced aging model, oral administration of oleuropein resulted in a dose-dependent reduction in pro-inflammatory cytokines concentrations, including IL-1β and TNF-α, highlighting its potential role in mitigating age-related neuroinflammation [318]. In another experimental model of brain injury following renal ischemia–reperfusion, treatment with oleuropein (100–200 mg/kg) decreased IL-1β, TNF-α, and nuclear Factor kappa-light-chain-enhancer of activated B cells p65 subunit (NF-κB-p65) levels while increasing anti-inflammatory IL-10 and Nrf2 expression; no effects were observed at 50 mg/kg [319]. In male Wistar rats with morphine-induced hippocampal neurotoxicity and memory deficits, treatment with oleuropein (15 and 30 mg/kg) improved antioxidant enzyme activity, including SOD and GPx, and ameliorated memory deficits and spatial learning impairments. Moreover, oleuropein reduced lipid peroxidation and neuronal apoptosis by regulating the expression of Bcl-2/Bax (Bcl-2-associated X protein) proteins [320].

Mechanistically, oleuropein has been shown to inhibit the formation of toxic Aβ aggregates and to reduce neuroinflammation and mitochondrial ROS/RNS production [321,322]. Furthermore, several studies have suggested a protective and preventive role of oleuropein in PD through inhibition of α-synuclein-induced toxicity in dopaminergic neurons [323]. Currently, there are no clinical studies evaluating oleuropein alone for brain health (Table 6).

### 9.2. Hydroxytyrosol

HT, a major olive polyphenol, exerts neuroprotective effects partly through activation of the Keap1–Nrf2–ARE signaling pathway, which enhances endogenous antioxidant defenses and reduces oxidative stress and neuroinflammation. These mechanisms may contribute to protection against cognitive decline and neurodegenerative processes associated with brain aging [325,326]. Experimental studies further support these effects. In a rotenone-induced PD model, HT-rich olive polyphenols reduced oxidative stress, neuroinflammation, and dopaminergic neuronal damage [327]. In another study, HT attenuated oxidative stress and neuroinflammation and improved depressive-like behavior in mice by activating the BDNF/TrkB/CREB signaling pathway in the hippocampus [328].

Clinical evidence has also begun to emerge. In an RCT, participants received 3 g of dried olive tree polyphenols, containing a high concentration of HT (16.2 mg/g), or placebo in olive oil twice daily for 12 weeks. Among the evaluated cognitive domains, complex attention showed a significant time × group interaction between the dried olive tree polyphenols and placebo groups. Significant time-related improvements were also observed in psychomotor speed, reaction time, cognitive flexibility, processing speed, and executive function [324]. In another RCT involving patients with AD, daily consumption of a beverage prepared from Greek olive leaves was associated with improvements in cognitive performance and reductions in oxidative stress markers, supporting the potential neuroprotective effects of olive polyphenols [329].

## 10. Limitations of the Study and Further Research

This review was not intended to be exhaustive; the relevant literature is extensive, heterogeneous in design and endpoints, and rapidly evolving. Herein we focused our search on studies situated at the higher levels of evidence, such as RCTs, systematic reviews, and meta-analyses. Nonetheless, many studies involved small samples and were most likely, underpowered. Consequently, in many instances, only within-group changes were reported because between-group differences could not be established. In the absence of significant between-group differences, these changes may reflect nonspecific effects and should be assessed accordingly.

In addition, although preclinical and clinical findings are presented in parallel, important discrepancies between these levels of evidence should be acknowledged. Several compounds have demonstrated robust mechanistic efficacy in in vitro and animal models; however, they have failed to translate into consistent clinical benefits in humans. This translational gap may reflect differences in dosing, bioavailability, and pharmacokinetics, as well as the limited ability of preclinical models to fully replicate the complexity of human neurodegenerative diseases. Thus, caution is warranted when extrapolating mechanistic findings to clinical efficacy, and further translational studies are needed to better align experimental models with clinical outcomes.

We deliberately excluded studies that evaluated multicomponent formulations because it would be difficult to attribute the observed benefits to any single compound. However, this approach may also overlook potential synergistic or additive effects, which are increasingly recognized in nutraceutical research, particularly in combinations targeting multiple pathways such as neuroinflammation, oxidative stress, and neurotransmission. Another caveat was the potential for language bias; we acknowledge an underrepresentation of non-English studies, which may have affected the level of evidence presented for compounds that are more widely used in Asia (i.e., Lion’s Mane). Notably, we identified several very recent additions to the literature from Europe [150,202], which may indicate an emerging shift in the available evidence in the following years.

Future research should prioritize well-designed, adequately powered, multicenter randomized controlled trials with standardized dosing regimens, longer follow-up periods, and harmonized clinical endpoints. Importantly, these studies should incorporate mechanistic biomarkers (e.g., NF-κB, NRF2, BDNF/TrkB signaling, amyloid, and tau markers) to strengthen causal inference and bridge the gap between preclinical findings and clinical efficacy. In parallel, stratified or precision-medicine approaches should be implemented to better define responders, taking into account genetic, biological, and metabolic variability.

Genetic polymorphisms are key determinants of treatment response. For instance, variants in the *MTHFR* gene can alter folate metabolism, leading to elevated homocysteine levels and impaired methylation processes associated with cognitive decline and neuropsychiatric disorders [330,331]. Emerging human data further support the role of one-carbon metabolism-related gene variants in modulating folate status and cognitive outcomes, underscoring the importance of the genetic background in therapeutic efficacy [332]. In addition to host genetics, the gut microbiome is another critical modulator, particularly for compounds such as polyphenols and flavonoids. As only a small proportion (5–10%) of dietary polyphenols is directly absorbed, most undergo microbial biotransformation into bioactive metabolites, making microbiota composition a key driver of bioavailability and efficacy [333]. Furthermore, metabolic factors, including age, insulin resistance, systemic inflammation, and epigenetic regulation, can further influence treatment outcomes. For example, polyphenols partly exert their neuroprotective effects through modulation of redox-sensitive pathways such as Nrf2, which is itself subject to epigenetic regulation and inter-individual variability, thereby affecting antioxidant capacity and neuroinflammatory responses [12].

Finally, given the growing interest in these compounds, future investigations should also explore advanced delivery systems (e.g., nanoparticle carriers, phospholipid complexes, liposomal formulations, and controlled-release technologies) to enhance bioavailability and central nervous system penetration, thereby improving their translational potential.

## 11. Conclusions

There is increasing evidence that nutraceuticals can exert beneficial effects on brain health. Among these, Huperzine A, flavonoids, and olive oil polyphenols are the most promising candidates. Huperzine A demonstrates robust cholinesterase inhibition, antioxidant, and neuroprotective effects, with supporting clinical evidence for cognitive improvement. Flavonoids, particularly luteolin, quercetin, and apigenin, exhibit multi-target neuroprotection by modulating oxidative stress, neuroinflammation, microglial activation, and neurotrophic signaling pathways, such as BDNF/TrkB/CREB. Luteolin, in particular, combines potent anti-inflammatory and antioxidant effects with emerging clinical support in neurodevelopmental and neuropsychiatric conditions, highlighting its translational potential. Olive oil polyphenols, including oleuropein and hydroxytyrosol, provide complementary neuroprotective actions by regulating oxidative stress, neuro-immune tone, and mitochondrial function, with preliminary evidence for cognitive benefits in humans. Other compounds, such as Lion’s mane, biotin, folinic acid, and PEA, show encouraging safety profiles and preliminary mechanistic or preclinical efficacy; however, clinical validation remains limited.

Nutraceuticals are cost-effective, complementary alternatives to conventional treatments for various brain-related conditions. A major advantage of these agents is their favorable safety profile, which allows for long-term use, particularly for prevention. Safety and efficacy are highly compound- and dose-dependent, and robust conclusions require adequately powered, well-designed clinical trials with long-term follow-up periods.

## Figures and Tables

**Table 2:** Preclinical and clinical effects of folic acid.

Mechanism/ Target	Preclinical Effects	Clinical Effects	References
↓ Homocysteine, ↑ SAM, ↑ DNA/histone methylation; ↓ oxidative stress; ↓ pro-inflammatory cytokines	Restored oxidative stress in LPS neuroinflammation; improved memory & learning; attenuated Aβ and tau pathology; improved PD motor symptoms; reduced neuronal death in ischemia; antidepressant effects via ↑ monoamines, BDNF, β-endorphin	In PD: low plasma folate in patients with cognitive impairment; B vitamin co-supplementation ↓ Hcy in levodopa-treated patients. In MCI and AD: folic acid ± B12 ↓ Hcy, ↑ SAM, ↓ IL-6/TNF-α/Aβ42, ↑ cognitive scores. Stroke: ↓ risk by 10%, improved recanalization outcomes. Depression: adjunctive methylfolate improves symptoms	[85,86,97,98,99,100]

AD: Alzheimer’s disease; BDNF: brain-derived neurotrophic factor; Hcy: homocysteine; LPS: Lipopolysaccharide; PD: Parkinson’s disease; SAM: S-adenosylmethionine; TNF-α: tumor necrosis factor-alpha; ↑: increased; ↓: decreased.

**Table 3:** Clinical effects of Palmitoylethanolamide (PEA).

Compound	Model/Population	N	Dosage	Mechanism of Action	Key Outcomes	Reference
PEA (Levagen+)	Healthy university students	39	Formulated PEA ~700 mg/day	↑ serum BDNF; improves memory	↑ BDNF, improved memory outcomes	[151]
PEA (Levagen+)	Healthy female students	16	Formulated PEA ~700 mg/day	Modulates physiological stress	Improved heart rate variability and stress markers	[152]
PEA	Patients with sleep latency issues	103	PEA 300–600 mg/day	Neuromodulatory	↓ time to fall asleep, improved cognitive function upon waking	[153]
PEA (ultramicronized)	Adults with MDD	58	PEA 600 BID	Anti-inflammatory, endocannabinoid modulation	↑ response rates, ↓ depressive symptoms	[156]
PEA (ultramicronized)	Patients in acute mania (adjunct to lithium + risperidone)	70	PEA 600 BID	Anti-inflammatory, neuromodulatory	Improved manic symptoms and overall clinical status	[157]
PEA (ultramicronized)	Patients with primary negative schizophrenia	60	PEA 600 BID	Anti-inflammatory, neuromodulatory	Improved symptom scores when combined with risperidone	[158]

BDNF: brain-derived neurotrophic factor; BID: twice a day; MDD: major depressive disorder; PEA: palmitoylethanolamide; ↑: increased; ↓: decreased.

**Table 6:** Olive oil polyphenols: preclinical and clinical evidence, mechanisms, and outcomes.

Compound	Mechanism/Target	Preclinical Effects	Clinical Effects	References
Oleuropein	Crosses BBB; inhibits Aβ aggregation, ↓ neuroinflammation (IL-1β, TNF-α), ↑ antioxidant enzymes (SOD, GPx)	Reduced neuroinflammation, oxidative stress, apoptosis in TBI, morphine-induced toxicity, renal ischemia models; protective in PD and AD models	N/A	[315,320,321]
HT	Activates KEAP1/NRF2/ARE, modulates microglia, ↑ BDNF/TrkB/CREB, antioxidant & anti-inflammatory	↓ oxidative stress and dopaminergic neuron damage in PD; ↓ neuroinflammation & depressive-like behavior in mice	RCT: 3 g/day dried olive polyphenols (16.2 mg/g HT) for 12 wks improved attention, psychomotor speed, reaction time, executive function; Greek olive leaf beverage improved cognition and ↓ oxidative stress in AD patients	[314,324]

Aβ: amyloid-beta; AD: Alzheimer’s disease; ARE: Antioxidant Response Element; BBB: blood–brain barrier; BDNF: brain-derived neurotrophic factor; CREB: cAMP response element-binding protein; GPx: glutathione peroxidase; HT: hydroxytyrosol; IL-1β: interleukin-1 beta; KEAP1: Kelch-like ECH-associated protein 1; N/A: not applicable; NRF2: Nuclear factor erythroid 2-related factor 2; PD: Parkinson’s disease; SOD: superoxide dismutase; TBI: traumatic brain injury; TNF-α: tumor necrosis factor alpha; TrkB: tropomyosin receptor kinase B; wks: weeks; ↑: increased; ↓: decreased.

## Data Availability

No new data were created or analyzed in this study. Data sharing is not applicable to this article.

## Abbreviations

The following abbreviations are used in this manuscript:

2-IB	2-iminobiotin
5-MTHF	5-methyltetrahydrofolate
5,10-Methylene-THF	5,10-methylenetetrahydrofolate
6-OHDA	6-hydroxydopamine
ACC	Acetyl-CoA Carboxylase
ACE	Addenbrooke’s Cognitive Examination
ACE2	Angiotensin-converting enzyme 2
ACh	Acetylcholine
AChE	Acetylcholinesterase
AD	Alzheimer’s disease
ADHD	Attention-deficit hyperactivity disorder
Akt	Protein Kinase beta
AlCl_3_	Aluminum chloride
ALS	Amyotrophic lateral sclerosis
AP-1	Activator Protein 1
APP	Amyloid precursor protein
ARE	Antioxidant Response Element
Aβ	Amyloid beta
ASD	Autism spectrum disorder
BACE1	Beta-site APP cleaving enzyme 1
BBB	Blood-brain barrier
BBGD	Biotin-responsive basal ganglia disease
BCCP	biotin carboxyl carrier protein
Bcl2	B-cell lymphoma 2
BDI	Beck Depression Inventory
BDNF	brain-derived neurotrophic factor
BH4	tetrahydro-biopterin
BID	Twice daily
BV-2	Brain 2
CASI	Cognitive Abilities Screening Instrument
cAMP	Cyclic Adenosine Monophosphate
CAT	Catalase
CB	Cannabinoid receptors
CDR	Clinical Dementia Rating
c-FOS	cellular Finkel-Biskis-Jinkins murine osteogenic sarcoma virus
CNS	Central nervous system
COMPASS	Computerized mental performance assessment system
COMT	Catechol-O-methyltransferase
COX-2	Cyclo-oxygenase-2
CREB	cAMP response element-binding protein
CRP	C-reactive protein
CSD	cortical spreading depression
CVD	Cardiovascular disease
CVLT-II	California Verbal Learning Test-II
DALYs	Disability-adjusted life years
DNA	Deoxyribonucleic acid
EAHE	A-enriched *Hericium erinaceus*
ERK	Extracellular signal-regulated kinase
EU	European Union
FAAH	Fatty acid amide amidohydrolase
FTD	Frontotemporal dementia
FRA	Folate receptor autoantibodies
GABA	Gamma-aminobutyric acid
GDNF	Glial cell line-derived neurotrophic factor
GFAP	Glial fibrillary acidic protein
GPR119	G protein-coupled receptor 119
GPR55	G protein-coupled receptor 55
GPx	Glutathione peroxidase
GR	Glutathione reductase
GRP78	Glucose-related protein 78
GSH	glutathione
GSK-3	Glycogen Synthase Kinase-3
GSSG	Glutathione disulfid
GST	glutathione-S-transferase
HAT	Histone acetyltransferase
Hcy	Homocysteine
HDAC	Histone Deacetylase
HE	*Hericium erinaceus*
HEM	*Hericium erinaceus* mycelia
HEME	*Hericium erinaceus* mycelial extracts
HLCS	holocarboxylase synthetase
HO-1	Heme oxygenase-1
HPA	Hypothalamic–pituitary–adrenal
HT	Hydroxytyrosol
HupA	*Huperzine A*
IADL	Instrumental Activities of Daily Living
IGAP	Intersectoral Global Action Plan on Epilepsy and Other Neurological Disorders
IL-1α/β	Interleukin 1α/β
IL-6	Interleukin-6
iNOS	Inducible Nitric Oxide Synthase
IRE1α	Inositol-requiring transmembrane kinase/endoribonuclease 1α
IVIG	Intravenous immunoglobulin
JNK	c-Jun N-terminal kinases
KEAP1	Kelch-like ECH-associated protein 1
LPS	Lipopolysaccharide
MAPK	Mitogen-Activated Protein Kinase
MCC	Methylcrotonyl-CoA carboxylase
MCI	Mild cognitive impairement
MDA	malondialdehyde
MMSE	Mini-Mental State Examination
MPO	Myeloperoxidase
MPTP	1-methyl-4-phenyl-1,2,3,6-tetrahydropyridine
mRNA	messenger Ribonucleic Acid
MS	Multiple sclerosis
MTHFR	Methylenetetrahydrofolate reductase
NAPE	N-acylphosphatidylethanolamine
NF-κB	Nuclear Factor kappa-light-chain-enhancer of activated B cells
NF-κB-p65	Nuclear Factor kappa-light-chain-enhancer of activated B cells p65 subunit
NGF	Nerve growth factor
NLR	Neutrophil-to-lymphocyte ratio
NLRP3	NLR family pyrin domain containing 3
NMDA	N-Methyl-D-aspartic acid
NOS	Nitric oxide synthase
NEG2	Nuclear factor erythroid 2-related factor 2
NTDs	Neural tube defects
PC	Pyruvate Carboxylase
PCC	Propionyl-CoA Carboxylase
PD	Parkinson’s disease
PEA	Palmitoylethanolamide
PEALut	PEA and luteolin
PI3K	Phosphatidylinositol-3-kinase
PKC	Protein Kinase C
PNEI	Psycho-neuro-endocrino-immunology
PPAR-α	proliferator-activated alpha
PPAR-γ	proliferator-activated gamma
PPMS	Primary progressive MS
PP2A	Protein phosphatase 2A
PS1	Presenilin 1
PTEN	Phosphatase and TENsin homolog
RCT	Randomized controlled trial
RNS	Reactive nitrogen species
ROS	Reactive oxygen species
RRMS	Relapsing–remitting multiple sclerosis
SAM	S-adenosylmethionine
SOD	superoxide dismutase
SPMS	Secondary progressive multiple sclerosis
TBI	Traumatic brain injury
THF	Tetrahydrofolate
TLR-4	Toll-like receptor 4
TNF-α	Tumor necrosis factor α
TrkB	Tropomyosin receptor kinase
UK	United Kingdom
VAS	Visual analogue scale
WHO	World Health Organization
β-CTF	β-secretase-derived carboxy-terminal fragment
